# State Laws Matter When It Comes to District Policymaking Relative to the Whole School, Whole Community, Whole Child Framework

**DOI:** 10.1111/josh.12959

**Published:** 2020-11-12

**Authors:** Jamie F. Chriqui, Julien Leider, Deborah Temkin, Elizabeth Piekarz‐Porter, Rebecca M. Schermbeck, Victoria Stuart‐Cassel

**Affiliations:** ^1^ Professor, (jchriqui@uic.edu), Division of Health Policy and Administration and Institute for Health Research and Policy School of Public Health, University of Illinois Chicago, 1603 W. Taylor Street, M/C 923, Chicago, IL 60612.; ^2^ Senior Research Specialist, (jleide2@uic.edu), Institute for Health Research and Policy University of Illinois Chicago, 1747 W. Roosevelt Road, M/C 275, Chicago, IL 60608.; ^3^ Vice‐President for Youth Development and Education Research, (dtemkin@childtrends.org) Child Trends, 7315 Wisconsin Avenue, Suite 1200W, Bethesda, MD 20814.; ^4^ Clinical Assistant Professor, (epiekarz@uic.edu), Division of Health Policy and Administration School of Public Health, University of Illinois Chicago, 1603 W. Taylor Street, M/C 923, Chicago, IL 60612.; ^5^ Research Specialist, (rscherm@uic.edu), Institute for Health Research and Policy University of Illinois Chicago, Chicago, IL 60608.; ^6^ President, (tori@emt.org), EMT Associates, Inc., 1631 Creekside Drive, Suite 100, Folsom, CA 95630.

**Keywords:** school health, legal epidemiology, child health, education, WSCC model, school health policy

## Abstract

**BACKGROUND:**

The Whole School, Whole Community, Whole Child (WSCC) framework supports the “whole child” across 10 domains. This study assessed state law and district policy WSCC coverage.

**METHODS:**

Primary legal research was used to compile relevant district policies and state laws for a stratified random sample of 368 public school districts across 20 states for school year 2017‐18. Policies/laws were evaluated on 79 items across the WSCC domains (range: 3‐14 items/domain). Multivariable regressions examined the relationship between state laws and district policies, controlling for district characteristics, and weighted to account for the sample design and non‐response.

**RESULTS:**

On average, district policies and state laws addressed 53% and 60% of the 79 items, respectively. State law predicted district policy WSCC attention across items (coeff. = 0.26, 95% CI = 0.14, 0.38) and 4 domains: physical activity (coeff. = 0.57, 95% CI = 0.29, 0.86); health services (coeff. = 0.50, 95% CI = 0.39, 0.62); social and emotional climate (coeff. = 0.34, 95% CI = 0.23, 0.45); and family engagement (coeff. = 0.41, 95% CI = 0.28, 0.54). State law was associated with lower district‐level coverage in 3 domains (health education; counseling, psychological, and social services; and community involvement).

**CONCLUSIONS:**

Although WSCC implementation is locally‐driven, states have an active role to play in setting a policy “floor” for guiding district WSCC attention.

The Whole School, Whole Community, Whole Child (WSCC) framework was created by Association for Supervision and Curriculum Development (ASCD) and the Centers for Disease Control and Prevention (CDC) to provide an integrated approach to improving student health and educational outcomes,^1^, ^2^, ^3^, ^4^ and to focus on supporting and engaging the whole child.^2^, ^3^, ^5^, ^6^, ^7^ WSCC is a 10‐component framework that links child and school health and wellness (health education, nutrition education and services, physical activity (PA) and physical education (PE), and health services) with behavioral/psychosocial supports (social and emotional climate; counseling, psychological, and social services), the physical school environment, employee wellness, family engagement, and community engagement.

Although WSCC is intended to guide local, school‐level support of the whole child, policies governing school practices are made at the state and school district levels. State policymakers enact statutes and regulations with the assumption that these recommendations and mandates will translate into district and school practice^8^ and, in other cases, districts adopt policies that exceed state law provisions or in areas where state law is silent to reflect their circumstances. While on‐the‐books laws and policies do not equate to implementation in practice, understanding the universe of such laws and policies is a critical first step for states and districts to monitor compliance. At the same time, except for studies within discrete policy areas such as nutrition, physical education, and bullying,^9^, ^10^, ^11^ little is known about whether state law attention results in increased attention at the district level. Understanding whether state law attention is associated with corresponding district policy attention is critical to inform how best to encourage implementation of strategies supportive of the whole child.

To date, there have been studies of policy actions at the state and/or district levels within specific WSCC domains including, but not limited to, health education;^12^ health services;^13^, ^14^, ^15^ nutrition environment and services, PE/PA, and employee wellness;^16^, ^17^, ^18^, ^19^ social and emotional climate;^20^, ^21^, ^22^ and the physical environment domain (related to school discipline).^23^ And, 2 recent reports provided the first nationwide assessments of WSCC‐related statutes and regulations across the 50 states and the District of Columbia (DC) as well as an in‐depth assessment of WSCC‐related district policies.^16^, ^24^ These latter reports provide the foundation for the current study.

Yet, to the best of our knowledge, no published peer‐reviewed study has explored whether, and in what domains, the content of state laws is associated with the content of district policies related to the WSCC. This study seeks to fill this gap. We hypothesize that greater attention to topics in state law will be associated with greater attention in district policies across WSCC domains.

## METHODS

### Sample

A stratified random sample of 399 public school districts across 20 purposefully selected states was obtained from the National Center for Education Statistics' (NCES) 2014‐2015 Public Elementary/Secondary School Universe Survey.^25^ The 20 states (inclusive of DC) were strategically selected to reflect a range of characteristics related to WSCC and children's health and well‐being: the four most populous states (California, Florida, New York, and Texas); childhood obesity rates (highest: Mississippi and South Carolina; lowest: New Jersey and Oregon); bullying rates (highest: Idaho and Nebraska; lowest: D.C. and Rhode Island); chronic absenteeism rates (highest: Alaska and Washington; lowest: Indiana and North Dakota); and four states purposefully selected as part of the Robert Wood Johnson Foundation's Together for Healthy and Successful Schools Initiative (Colorado, Michigan, Missouri, and New Mexico). Since some states fell into multiple categories such as the highest or lowest childhood obesity and absenteeism rates, the next ranked state not already represented was chosen for inclusion.

Using the NCES CCD data,^25^ public school district and charter local education agencies (LEAs) were stratified into sextiles of student population size. Due to the limited online policy availability for very small LEAs, LEAs in the bottom sextile were excluded. The LEAs in the top 5 sextiles were grouped into within‐state strata by family income level (based on the proportion of students eligible for free and reduced‐price lunch [FRPL]), student diversity (race/ethnicity of district students) as measured through Simpson's Diversity Index,^26^ and urbanicity (urban/rural). Twenty‐four LEAs were sampled for each state, with proportional representation between public and charter LEAs. For purposes of this paper, we focus specifically on public school districts. Ultimately, 399 public school districts were sampled across the 20 states.

### Procedures

#### *Policy collection*

District policies and state laws effective as of the day after Labor Day 2017 (proxy for the beginning of school year 2017‐18) were compiled by trained collectors. Detailed descriptions of the methods for compiling the district policies and state laws are provided elsewhere; we briefly describe the methods herein.^16^, ^24^

For the district policies, a collection protocol was developed that built upon prior nationwide district policy collection efforts from the National Wellness Policy Study^18^ and the topics included in the coding protocol described below. The collection protocol guided the systematic collection of the district policies across the 10 WSCC domains. “Policy” was defined to include school board policies, superintendent regulations, and relevant student handbooks that included provisions related to school discipline and physical environment‐related policies. Relevant policies were able to be obtained for 368 of the 399 sample districts via Internet research (with telephone and electronic mail follow‐up as necessary) by 2 of the study authors and trained, Master's level research assistants (RAs); policies were not able to be obtained for the remaining 31 districts. The policy collection rate (92%) was comparable to prior nationally representative district policy collection efforts (range 94%‐97%).^18^, ^19^

State laws for the 20 states were obtained through primary legal research conducted using the codified state statutory and regulatory databases available by subscription from LexisNexis and Westlaw.^27^, ^28^ The state databases were searched using Boolean search strings to capture relevant statutes and regulations across each of the 10 domains. State laws were compiled by two of the study authors and a JD‐level RA. Where possible, the state laws were validated against existing secondary sources of relevant state laws.^17^, ^22^, ^29^, ^30^, ^31^, ^32^, ^33^, ^34^, ^35^, ^36^, ^37^, ^38^, ^39^

It should be noted that the DC has only one public school district (DC Public Schools). State laws encompass statutes and regulations passed by the DC Legislative Council and the DC State Board of Education, which apply to both public schools and public charter schools (the latter of which are not included for the current sample). District policies for DC include only those administered by DC Public Schools (along with state laws embedded by reference).

#### *Policy coding*

Health Education (Appendix S1) provides the detailed coding guide. The coding scheme built upon prior state law and/or district policy coding schemes on relevant topics as well as best practices and national standards and guidelines from authoritative and governmental bodies.^22^, ^30^, ^33^, ^40^, ^41^, ^42^, ^43^, ^44^, ^45^, ^46^, ^47^, ^48^, ^49^, ^50^, ^51^, ^52^, ^53^, ^54^, ^55^, ^56^, ^57^, ^58^, ^59^, ^60^, ^61^, ^62^, ^63^, ^64^, ^65^, ^66^, ^67^, ^68^, ^69^, ^70^, ^71^ Trained attorneys and master's‐level coders at the Institute for Health Research and Policy at the University of Illinois Chicago (UIC) and EMT Associates, Inc. coded the state law data. Two of the study authors led the state law coding teams at UIC and EMT Associates, respectively. All coders were trained on the coding scheme and inter‐coder reliability was computed. Since the state law coding was split across institutions by domain (UIC and EMT both coded 5 domains in their entirety so there was no overlap of coding within domains), the reliability coding was institutionally‐based. At UIC, the state law coding was conducted by the master coder and a study author and another coder, both of whom have JD degrees. Reliability testing was conducted on the first 25 states coded, with an overall percent agreement of 89.66% and an inter‐coder agreement of κ = 0.83 which is considered to be a high level of agreement.^72^ At EMT Associates, the state law coding was conducted by the master coder and study author and a second coder. Reliability testing was conducted on the first 20 states coded, with an overall percent agreement of 86.07% and an inter‐coder agreement of κ = 0.79, which is considered as a moderately‐strong level of agreement.^72^ Once training and reliability coding was completed, all state laws were double‐coded and reviewed to form a consensus coding for each variable within each state.

The district policy coding was conducted by UIC. Given the volume of districts and policies within each state and similarities in policies across districts within a state, district policy coding was conducted on a state‐by‐state basis by coders. For example, all California districts were coded by one coding team. For training purposes, 2 public school districts within each state were double‐coded and reviewed by a master coder and study author for consistency. Given that many school districts within the same state adopt model policies issued by the state Board of Education or state school board association, any applicable codes were applied by the first coder to all such policies and districts. Any unique, non‐model, policies were then double‐coded and reviewed by both coders to reach a consensus coding. All coding teams included one of the two master coders to ensure coding consistency. In addition, many districts embed state law language by reference to specific state statutory or regulatory citations within the body of their policies. In such instances, the appropriate state law coding was applied.

### Data Analysis

#### *Recoding of policy data and summary score computation*

For purposes of this study, all state law and district policy data were recoded into binary (yes/no) measures of whether the given variable was addressed or not. For grade‐specific items regarding health education, health services, PE time, and nutrition services, the addressed measures were computed based on whether the given item was addressed for any grade level.

In addition, the binary measures for some variables needed to be tailored to the unique nature of the item. For example, within the physical environment domain, the original coding scheme for corporal punishment was: 0 (permitted), 1 (silent on whether corporal punishment was permitted or prohibited), and 2 (prohibited). Given that prohibiting corporal punishment is supportive of the “whole child” while being silent on the issue is subject to interpretation, for purposes of this analysis, a binary variable = 1 (addressed) was created to reflect only cases where corporal punishment was prohibited rather than also including cases where the law/policy was silent on the issue.

In other instances, more than one variable captured a construct. For example, in the PE/PA domain, 2 separate items were coded regarding sports participation fees—whether fees were allowed or prohibited; a single “addressed” measure was computed from these 2 variables indicating whether fees were either prohibited or allowed but with waivers. Similarly, in the nutrition environment and services domain, 2 items captured unpaid meal charges (provisions that supported students such as not singling them out and provisions that restricted access such as only providing a sandwich and milk to students with unpaid meal charges). For this analysis, unpaid meal charges were only counted as addressed where a policy was in place to support students.

The binary measures for each variable within a given domain were summed and divided by the total number of measures in the domain to create a domain‐specific summary score for each state and district. Similarly, all 79 binary measures used herein were summed and divided by 79 to create an overall summary score for each state and district.

#### *Weighting of the data*

Details on the district weighting are reported elsewhere.^24^ Briefly, district weights were computed by state and sampling stratum (defined by family income level, student diversity, and urbanicity, as discussed earlier) with the goal of weighting the districts in the final sample to reflect the total number of public school districts within each state and sampling stratum.

#### *Statistical analysis of the policy data*

Using svy commands in Stata/SE 13.1 accounting for sampling strata and the weights described above (with scaling to account for strata with a single sampling unit), descriptive statistics were computed for the mean prevalence of items addressed within each domain and across all domains and all districts. The state law data represent a census of the 20 states' data and, therefore, were unweighted.

To assess the extent to which state law predicted district policy attention to the various domains and overall, unadjusted and adjusted linear regressions were computed using svy commands in Stata/SE 13.1. The unadjusted models included the state summary score for each domain (or overall) as the predictor and the district policy summary score for each domain (or overall) as the outcome. The adjusted models added in controls for majority race/ethnicity of the districts' students, FRPL eligibility, urbanicity/locale, district size, and Census region. Finally, Appendix S2 models 2 scenarios: the predicted change in district policy scores by domain and overall if (1) the state law score was at the mean for the given domain or overall, and (2) if the state law fully covered the domain or all 79 items examined for this study.

## RESULTS

Across the 368 districts, 28,978 potentially relevant school board policies, regulations, and handbooks (collectively referred to as “policies” hereafter) were collected, with an average of 78.7 policies per district. Table 1 presents the weighted district characteristics. The majority of the districts (55%) had a majority white (≥66%) student population and another quarter (26%) had a diverse (no racial/ethnic majority) student population. The districts were relatively evenly distributed across FRPL tertiles (which was expected given the sampling strategy). Only 7% of the districts were in large to mid‐size cities; the rest were in suburban, rural, or township areas. Most districts (57%) were considered large based on student enrollment and the districts were relatively evenly distributed across Census regions.

**Table 1:** Weighted District Sample Characteristics

Characteristic	% of Districts
Majority race/ethnicity of students	
White (≥66% white)	55
Black (≥50% Black)	4
Hispanic (≥50% Hispanic)	15
Diverse (no majority race/ethnicity)	26
Proportion of students eligible for free/reduced‐price lunch†	
Low (High‐Income) (0‐38.54%)	35
Medium (>38.54%‐60.83%)	32
High (Low‐Income) (>60.83%‐100%)	33
Locale	
Large to mid‐size city	7
Suburban	32
Rural	42
Township	19
District size (based on student enrollment)	
Small (244‐403 students)	11
Medium (404‐1540)	32
Large (1541‐207,469)	57
Census region	
West	28
Midwest	27
South	22
Northeast	22

Note. N = 368 districts.

† Only 355 districts had free/reduced‐price lunch data.

Table 2 presents the prevalence of items addressed within each WSCC domain and overall for the state laws and district policies. On average, state laws addressed at least one‐half to three‐fourths of all of the items evaluated in 8 of the 10 domains (the only domains where state law minimally addressed the domain were Nutrition Environment and Services and Employee Wellness). Across the district policies, at least one‐half to over three‐fourths of the items within a given domain were addressed on average in 7 of the 10 domains (the only exceptions being for Nutrition Environment and Services; Counseling, Psychological, and Social Services; and Employee Wellness). On average, state laws and district policies addressed 59.7% and 52.8%, respectively, of all items examined.

**Table 2:** Prevalence of Items Addressed in State Laws and District Policies by WSCC Domain

	Prevalence of Items Addressed^†^			
	State Laws		District Policies	
Domain (# Items)	%	Std. Dev.	%	95% CI
Community involvement (N = 3)	70.0	32.3	76.4	73.9, 79.0
Counseling, psychological, and social services (N = 8)	63.8	16.2	49.1	46.8, 51.5
Employee wellness (N = 5)	15.0	25.9	17.3	15.1, 19.5
Family engagement (N = 4)	60.0	27.4	53.5	51.0, 56.0
Health education (N = 10)	73.5	22.5	56.5	54.4, 58.7
Health services (N = 10)	69.0	22.5	52.9	51.0, 54.8
Nutrition environment and services (N = 6)	28.3	22.4	49.8	46.7, 52.9
Physical activity and education (N = 7)	51.4	20.9	56.6	53.2, 60.1
Physical environment (N = 12)	75.0	18.1	62.1	60.1, 64.0
Social and emotional climate (N = 14)	58.9	17.9	50.8	48.9, 52.7
*All items across all WSCC domains (N = 79)*	*59.7*	*14.4*	*52.8*	*51.7, 53.9*

CI, confidence interval; Std. Dev., standard deviation.

^†^ The state law columns represent the unweighted mean and standard deviation of the percent of items addressed within a given domain across the 20 states. The district policy columns represent the weighted mean percent of items addressed within the district‐level policies for a given domain across the 368 districts.

Results of the unadjusted and adjusted regression models where we examined the extent to which the scope of state law is associated with district policymaking across the domains and overall are presented in Table 3. (Appendix S2 presents the adjusted mean district policy scores for when the state law summary score was at the mean or full coverage for each domain and overall.) For brevity purposes, we only present the adjusted models in this discussion. State law was significantly associated with increased coverage in district policy in the overall model and in 4 domains (Family Engagement, Health Services, PE/PA, and Social and Emotional Climate). In other words, for each 1‐point increase in the percentage of items addressed within the domain (or overall) in state law, district policy coverage within the given domain (or overall) increased by the amount represented by the coefficient. For example, for overall coverage, with a 1‐point increase in the percentage of items addressed (out of 79 items) in state law, district policy coverage increased by 0.26 points (95% CI = 0.14, 0.38). Thus, in these domains, district policy attention corresponds to concomitant state law attention in the domain.

**Table 3:** Linear Regression Models Examining the Association between State Laws and District Policies by WSCC Domain

Domain	Unadjusted Models			Adjusted^‡^ Models		
	Coef.^†^	95% CI	p‐value	Coef.^†^	95% CI	p‐value
Community involvement	−0.08	−0.18, 0.01	.068	−0.13	−0.24, −0.02	.017
Counseling, psychological, and social services	−0.44	−0.57, −0.31	<.001	−0.23	−0.36, −0.10	.001
Employee wellness	0.14	−0.00, 0.28	.051	0.01	−0.13, 0.15	.877
Family engagement	0.03	−0.09, 0.14	.620	0.41	0.28, 0.54	<.001
Health education	−0.39	−0.48, −0.30	<.001	−0.74	−0.93, −0.55	<.001
Health services	0.56	0.46, 0.66	<.001	0.50	0.39, 0.62	<.001
Nutrition environment and services	0.10	−0.04, 0.24	.147	−0.05	−0.23, 0.13	0.608
Physical activity and education	0.90	0.72, 1.08	<.001	0.57	0.29, 0.86	<.001
Physical environment	0.07	−0.02, 0.17	.139	0.01	−0.12, 0.13	.912
Social and emotional climate	0.33	0.23, 0.43	<.001	0.34	0.23, 0.45	<.001
*Overall (all items)*	*0.12 *	*0.02, 0.21*	*.015*	*0.26 *	*0.14, 0.38*	*<.001 *

CI = confidence interval; Coef., coefficient.

† The coefficients represent the change in district policy scores within the given domain associated with a 1‐point increase in the percentage of items addressed in the state laws.

‡ Adjusted for characteristics noted in Table 1 (majority race/ethnicity of district students, free/reduced‐price lunch eligibility tertiles, urbanicity/locale, district size, and Census region). Adjusted models include 355 districts because free/reduced‐price lunch data were missing for 13 districts. Items in bold represent statistically significant associations between state laws and district policies at the p < .05 threshold or lower.

In contrast, in 3 domains (Community Involvement; Counseling, Psychological, and Social Services; and Health Education), state law coverage and district policy attention were inversely related. In other words, in these domains, when state law addressed more items within the domain, district policies addressed fewer items, and when state laws addressed fewer items district policies addressed more. For example, for each 1‐point increase in the percentage of items addressed in the Health Education domain in state law, district policy coverage decreased by 0.74 points (95% CI = −0.93, −0.55).

In the remaining 3 domains (Nutrition Environment and Services, Physical Environment, and Employee Wellness) there was not a significant association between state law and district policy content, indicating there was not a clear pattern across the sample of states.

## DISCUSSION

This study provides new insight into how state law content relates to district policies relevant to WSCC. Overall, state laws cover slightly more WSCC‐related provisions than district policies (59.7% vs. 52.8% on average, respectively) and, consistent with expectations, there is a significant positive association between state law and district policy coverage. As WSCC‐related provisions in state laws increase, so, too, do WSCC‐related provisions in district policy.

However, while this holds for the overall analysis, this pattern varies across the individual domains of the WSCC. The expected pattern—more district coverage as state coverage increases—holds for 4 domains: Family Engagement, Health Services, Physical Activity and Education, and Social and Emotional Climate. For these domains, state policy seems to have successfully translated down to district attention.

In 3 domains (Community Involvement; Counseling, Psychological, and Social Services; and Health Education), there is an inverse association with higher state law coverage associated with a lower rate of district policy coverage. It may be that the content of state laws precludes the need for districts to create explicit policy such as health education or PE standards. Alternatively, particularly for newer state laws, the influence of state policy may not yet have reached or been incorporated into district policy content. For example, descriptive analyses show that whereas 15 of the 20 states address professional development for suicide prevention (Counseling, Psychological, and Social Services), only 36% of districts did so.^24^ Given the recent policy push to include such provisions at the state level,^73^ it may be that the has been insufficient time for them to be reflected in district policy compared to other more long‐standing provisions.

Three domains (Nutrition Environment and Services, Physical Environment, and Employee Wellness) showed no clear associations between state law and district policy coverage. These findings should be considered in context: (1) our conceptualization of Nutrition Environment and Services for the present study purposefully did not include many provisions shown in previous studies to have widespread coverage at both the state and/or district levels;^17^, ^18^ (2) our conceptualization of the Physical Environment included items covering both physical security (such as restraint and seclusion) and environmental protections (such as air quality standards) which may have different patterns if separated; and, (3) few districts or states had any policies on employee wellness (consistent with prior research).^17^, ^18^

The inconsistent pattern in the relationship between state law and district policy coverage highlights the patchwork of approaches that states and districts have taken in law and policy across the WSCC domains. State laws covering the WSCC were all enacted at different times and may put competing requirements onto schools, and, as our analysis highlights, may have differential influence on district policies and related practices. Neither state laws nor district policies work in isolation from the other;^74^ for state laws to be successful at reforming school approaches to supporting students' social, emotional, and physical wellbeing, district policies must largely reflect the same content. State mandates do not work simply because policymakers believe they will; districts—and the schools they manage—must instead implement the intent of those laws with fidelity. The present analysis is a first step at understanding whether that process is working as intended.

As of school year 2017–2018, only 3 states' laws (DC, Vermont, and Washington) had explicitly addressed WSCC^16^ and 9% of districts in 20 states explicitly addressed WSCC in their policies.^24^ However, Coordinated School Health (CSH),^75^ the precursor to WSCC, has been widely addressed in state law and district policy.^16^, ^24^ Given the extensive overlap between CSH and WSCC,^75^ it is not surprising that jurisdictions have not specifically revised their laws and policies to mention WSCC by name because they already reference CSH by name; yet, what remains is a patchwork of policymaking related to WSCC nationwide.

### Limitations

The primary limitation of this study was that our analyzed laws and policies were from one school year (2017‐2018), and as such all of our findings are correlational in nature; neither directionality nor causality can be determined. Second, the district sample was drawn from 20 purposefully selected states using strata that accounted for a combination of FRPL eligibility rates, Simpson's Diversity Index, and urbanicity within each state. The district sample was not intended to be representative of the sociodemographic composition of all districts nationwide and, as such, some districts such as those with predominantly Black student populations may have been under‐sampled while those with diverse student populations may have been over‐sampled. Future studies would be well‐served to explore WSCC coverage across a nationally representative sample of districts and whether the association between state and district policies is different in a nationally representative study.

Third, we were unable to examine the implementation of the state laws and district policies. While there have been a plethora of studies conducted in the U.S. that examine the implementation and impact of state laws and/or district policies on a variety of school practices,^9^, ^76^, ^77^, ^78^, ^79^, ^80^, ^81^, ^82^, ^83^, ^84^, ^85^, ^86^, ^87^, ^88^, ^89^, ^90^, ^91^, ^92^, ^93^, ^94^, ^95^, ^96^, ^97^, ^98^ studies are needed that examine strategies for better coordinating implementation of state laws and district policies to support the whole child. This is an area for future research.

Future research also should address the combination of laws and policies that will be most effective at supporting the whole child. Relatedly, it is not clear that all of the policy provisions addressed herein are a “good thing.” As noted in methods, we reverse coded policy provisions such that we only counted provisions that would support students (such as only counting prohibitions on corporal punishment and only counting unpaid meal policies that did not identify or discriminate students). Given that there is significantly more literature on the implementation and/or impact of state laws and/or district policies in some WSCC domains (such as nutrition services and PE/PA) than other WSCC domains (such as social emotional climate), future research needs to determine which policy provisions are the most supportive of students.

Also, this study was based on an analysis of state laws and district policies from across 20 states; thus, findings reported herein may not be generalizable nationwide. However, the state sampling did capture the most populous states and states that reflect both high and low proportions of key WSCC‐related outcomes related to absenteeism, bullying, and obesity. Finally, while systematic methods were used to compile and code the state laws and district policies it is possible that a law or policy was over‐looked in the collection or coding process. We took steps to minimize missing relevant state laws through verification with secondary sources and through the use of systematic searches in commercial legal research databases for all states. And, for the state law coding, we achieved a high level of inter‐rater reliability. For the district policy collection, it is possible that policies were missed; however, given the sheer volume of district policies compiled (over 28,000 policies),^24^ missing policy information is not likely. In terms of district coding, all policies were double‐coded and a consensus coding was reached on all policies.

### Conclusions

In conclusion, this study found that both state laws and district policies address many elements of WSCC but neither do so comprehensively or holistically. While both states and districts make policies in this area, states have an active role to play in setting a policy “floor” for guiding district policy attention to WSCC and school‐level implementation and practices.

## Implications for School Health

As states amend their school health and education laws in the future, it should be with an eye toward supporting the whole child. Our findings demonstrate that the association between state laws and district policies may vary by policy topic and/or may take time to be reflected. Policymakers may want to consider taking a two‐pronged approach whereby WSCC‐related reforms are approached both from a “top‐down,” state law, approach as well as a “bottom‐up,” district policy, approach to ensure systems are aligned and there is buy‐in at all levels of the education system.

In addition, given the piecemeal nature of the current state policy landscape regarding WSCC, and its varied association with district policies, state policymakers may need to look to other opportunities to create a more holistic, statewide vision to support the whole child. One opportunity may come from states' approaches to implementing the federal *Every Student Succeeds Act (ESSA)* ^99^, which in 2015, amended the Elementary and Secondary Education Act of 1965. Under ESSA, states receive funding under Title‐IV Part A, the Student Success and Academic Enrichment Grant Program, to support student health and safety, the provision of a well‐rounded education (including physical and health education), and educational technology. A recent review of the states' ESSA plans indicated that 10 states specifically mentioned WSCC in their plan, with an additional 23 states' plans mentioning the “whole child.”^100^ Thus, states can use their authority under ESSA to take a whole child approach that supports the spirit behind WSCC—namely the integration of all of the components in support of the whole child.

Moving toward state laws and district policies that genuinely reflect a holistic vision of the WSCC will require considerable time and political will. Our analyses help provide a foundation regarding the current landscape of laws and policies, the associations between them, and opportunities for next steps.

### Conflict of Interest

The authors declare no conflicts of interest.

## Supporting information


**Appendix** **S1**. Whole School, Whole Community, Whole Child State and Local Policy Coding Rubric.

**Appendix** **S2**. Predicted Mean District Policy Score and Change in Score by Domain Associated with District‐Weighted Mean and Comprehensive State Law Scores
