# Supplementary material for: State Laws Matter When It Comes to District Policymaking Relative to the Whole School, Whole Community, Whole Child Framework
Source: J Sch Health. 2020 Nov 12;90(12):907–17. doi: 10.1111/josh.12959 (PMC7702124; doi:10.1111/josh.12959)
Supplement: Supplementary file 1 — Appendix S1. Whole School, Whole Community, Whole Child State and Local Policy Coding Rubric. [file JOSH-90-907-s001.pdf]

Appendix S1

Together for Healthy and Successful Schools
Whole School, Whole Community, Whole Child

State and Local Policy Coding Rubric

Developed by: Child Trends, EMT Associates, and the University of Illinois at Chicago

Version date: December 2018

Table of Contents

Health Education ... 3
Physical Activity and Physical Education ... 8
Nutrition Environment and Services ... 13
Health Services ... 19
Employee Wellness ... 26
Family Engagement ... 28
Community Involvement ... 31
Counseling, Psychological, and Social Services ... 34
Social and Emotional Climate ... 41
Physical Environment ... 57

Health Education

Variable		Domain	Measure	Score	Brief Description
**Health Education K-12 curriculum**	**HE1**	Curricula	Establishes a health education curriculum for **elementary** school students	**0**	Not addressed
				**1**	State law, regulation, or district policy recommends elementary HE curriculum, or requires HE for less than all years of elementary school<br><br>**Example(s)**<br>• *Health education should be an important part of the curriculum.*<br>• *Health education will be taught in 3-5 grades.*<br>• *Each school district may and is encouraged to establish a comprehensive health education program.*
				**2**	State law, regulation, or district policy requires elementary HE curriculum<br><br>**Example(s)**<br>• *There is established a K-8 health education curriculum.*<br>• *The content standards with benchmarks and performance standards for health education are mandated for students in grades K-12.*
	**HE2**		Establishes a health education curriculum for **middle** school students	**0**	Not addressed
				**1**	State law, regulation, or district policy recommends middle school HE curriculum, or requires HE for less than all years of middle school<br><br>**Example(s)**<br>• *Health education should be an important part of the curriculum.*<br>• *Health education will be taught for one semester in 7th grade.*<br>• *Each school district may and is encouraged to establish a comprehensive health education program.*
				**2**	State law, regulation, or district policy requires middle school HE curriculum<br><br>**Example(s)**<br>• *There is an established K-8 health education curriculum.*

Variable		Domain	Measure	Score	Brief Description
					• *The content standards with benchmarks and performance standards for health education are mandated for students in grades K-12.*
	**HE3**		Establishes a health education curriculum for **high** school students	**0**	Not addressed
				**1**	State law, regulation, or district policy recommends high school HE curriculum, or requires HE for less than all years of high school<br><br>**Example(s)**<br>• *Health education should be an important part of the curriculum.*<br>• *Health education will be taught for one semester in 9th grade.*<br>• *Each school district may and is encouraged to establish a comprehensive health education program.*
				**2**	State law, regulation, or district policy requires HE curriculum<br><br>**Example(s)**<br>• *There is an established health education curriculum for 9-12 grades.*<br>• *The content standards with benchmarks and performance standards for health education are mandated for students in grades K-12.*
	**HE1/2/3b**		Healthy eating/nutrition is taught as part of health education	**0**	Not addressed
				**1**	State law, regulation, or district policy addresses healthy eating/nutrition<br><br>**Example**<br>• *As part of a comprehensive health education program, pupils must be able to comprehend concepts related to the promotion of health and the prevention of disease to enhance health, as demonstrated by the ability to apply knowledge of food and nutrient needs when making decisions regarding food choices and meal plans.*
	**HE1/2/3c**		Mental and emotional health is taught as part of health education	**0**	Not addressed
				**1**	State law, regulation, or district policy addresses mental and emotional health

Variable		Domain	Measure	Score	Brief Description
					**Example**<br>• *Each school board shall consider adopting a distinct category on mental health instruction to educate pupils about all aspects of mental health. "Mental health instruction" shall include, but not be limited to reasonably designed and age-appropriate instruction on the overarching themes and core principles of mental health, defining common disorders, elucidating the services and supports, promoting mental health wellness.*
	**HE1/2/3d**		Personal health and wellness is taught as part of health education<br>(ex. hygiene, control of disease, cancer, self-exams)	**0**	Not addressed
				**1**	State law, regulation, or district policy addresses personal health and wellness<br><br>**Example**<br>• *Health education must include physical health and personal safety, CPR, cancer prevention and detection, dental health, family health, domestic violence, and the appropriate use of health services.*
	**HE1/2/3j**		Violence prevention is as part of health education	**0**	Not addressed
				**1**	State law, regulation, or district policy addresses violence prevention<br><br>**Example**<br>• *Each school district shall incorporate dating violence education that is age-appropriate into the school program. Dating violence education shall include, but not be limited to, defining dating violence, recognizing dating violence warning signs, and identifying characteristics of healthy dating relationships.*
	**HE1/2/3k**		Bullying prevention is taught as part of health education	**0**	Not addressed
				**1**	State law, regulation, or district policy addresses bullying prevention<br><br>**Example**<br>• *Health education shall include the development of healthy relationships. "Development of healthy relationships" includes, but is not limited to, all of the following: (1) understanding the principles of treating one another with respect, dignity, and kindness; (2) demonstrating the ability to use interpersonal*

Variable		Domain	Measure	Score	Brief Description
					communication skills to address and resolve disagreement and conflict; (3) recognizing when and how to respond to dangerous or other situations that may result in bullying, harassment, harming, or hurting of another person.
	**HE1/2/3l**		Suicide prevention is taught as part of health education	**0**	Not addressed
				**1**	State law, regulation, or district policy addresses suicide prevention<br><br>**Example**<br>• *The department of elementary and secondary education shall develop and prescribe a suicide prevention awareness program for public school students in grades 9 through 12. The school committees of the several cities, towns, and school districts shall provide for the incorporation of the program in existing health education courses.*
	**HE1/2/3m**		Social and emotional learning is taught as part of health education (includes self-awareness, interpersonal, working in groups, relationships, coping with life situations)	**0**	Not addressed
				**1**	State law, regulation, or district policy addresses social and emotional learning<br><br>**Example**<br>• *Social and emotional learning enhances students' capacity to integrate skills, attitudes, and behaviors to deal effectively and ethically with daily tasks. Health education will include social and emotional learning so that students are self-aware and make responsible decisions.*
**National HE Standards**	**HE4**	Standards	Addresses the National Health Education Standards either by name or through the adoption of individual standards	**0**	Not addressed
				**1**	State law, regulation, or district policy references National Health Education Standards as a guide for providing HE or includes a list of all National Health Education Standards without mentioning the standards by name<br><br>**Example**<br>*As part of health education, students will:*<br>• *Comprehend concepts related to health promotion and disease prevention to enhance health*<br>• *Analyze the influence of family, peers, culture, media, technology, and other factors on health behaviors*<br>• *Demonstrate the ability to access valid information,*

Variable		Domain	Measure	Score	Brief Description
					products, and services to enhance health<br>• *Demonstrate the ability to use interpersonal communication skills to enhance health and avoid or reduce health risks*<br>• *Demonstrate the ability to use decision-making skills to enhance health*<br>• *Demonstrate the ability to use goal-setting skills to enhance health*
**Health education professional development**	**HE7**	Staff qualifications and professional development	Professional development is provided to health education teachers	**0**	Not addressed
				**1**	State law, regulation, or district policy addresses providing professional development to all health education teachers<br><br>**Example**<br>• *Health education teachers will be provided with training to ensure that their methods and content are evidence-based.*<br>• *The Superintendent shall establish and maintain a system of teacher training that supports comprehensive school health education through appropriate staff development activities mandated for health and physical education classroom instructors.*

Physical Activity and Physical Education

Variable		Domain	Measure	Score	Brief Description
**National PE Standards**	**PA5**	Standards	References the National Physical Education Standards (SHAPE America) either by name or through the adoption of individual standards	**0**	Not addressed
				**1**	State law, regulation, or district policy addresses National Physical Education Standards as a guide for providing PE or includes a list of all National PE standards without mentioning the standards by name<br><br>**Example(s)**<br>• *All students will receive physical education in accordance with the Shape America Physical Education Standards.*<br>• *The State Board of Education shall ensure that the subject matter standards for physical education are consistent with national physical education standards for the information that students should learn about physical activity, and the physical activities that students should be able to perform.*
**Time for PE in elementary school**	**PA6**	Standards	Addresses the amount of time that is provided for physical education in **elementary** school every week, in accordance with National PE recommendations (150 min/week)	**0**	Not addressed
				**1**	State law, regulation, or district policy recommends that time for PE in elementary school that meets National Standards (150 min/week) or requires less than 150 minutes/week<br><br>**Example**<br>• *All students in elementary school will receive 150 minutes of physical activity per week. This will be accomplished through regular PE class and recess.* (time is split between recess and physical education)
				**2**	State law, regulation, or district policy requires that time for PE in elementary school meets National Standards (150 min/week)<br><br>**Example(s)**<br>• *Each district school board shall provide 150 minutes of physical education each week for students in kindergarten through grade 5.*<br>• *Physical education for elementary school students will meet National PE standards.*
**Time for PE in middle school**	**PA7**	Standards	Addresses the amount of time that is provided for physical education in **middle** school every week, in accordance with National PE recommendations (225 min/week)	**0**	Not addressed
				**1**	State law, regulation, or district policy recommends that time for PE in middle school that meets National Standards (225 min/week) or requires less than 225 min/week

Variable		Domain	Measure	Score	Brief Description
					**Example(s)**<br>• *Middle schools will strive to provide 225 minutes of physical education per week.*<br>• *The equivalent of one class period per day of physical education for one semester of each year is required for students enrolled in grades 6 through 8.*
				**2**	State law, regulation, or district policy requires that time for PE in middle school meets National Standards (225 min/week)<br><br>**Example(s)**<br>• *All students in middle school will receive 225 minutes of physical education per week.*<br>• *Physical education for middle school students will meet National PE standards*
**Time for PE in high school**	**PA8**	Standards	Addresses the amount of time that is provided for physical education in **high** school every week, in accordance with National PE recommendations (225 min/week)	**0**	Not addressed
				**1**	State law, regulation, or district policy recommends that time for PE in high school that meets National Standards (225 min/week) or requires less than 225 min/week<br><br>**Example**<br>• *High schools will strive to provide 225 minutes of physical education per week.*
				**2**	State law, regulation, or district policy requires that time for PE in high school meets National Standards (225 min/week)<br><br>**Example(s)**<br>• *All students in high school will receive 225 minutes of physical education per week.*<br>• *Physical education for high school students will meet National PE standards.*
**Adaptive PE**	**PA10**	Standards	Physical education is described as inclusive, including providing adapted physical education or accommodations when necessary for students.	**0**	Not addressed
				**1**	State law, regulation, or district policy addresses making PE inclusive, including adapted PE or accommodations when necessary<br><br>**Example**<br>• *A student with disabilities shall have suitably adapted*

Variable		Domain	Measure	Score	Brief Description
					physical education incorporated as part of the Individualized Education Program in order to meet physical education requirements. With a written note from a physician, public schools and public charter schools may provide suitably adapted physical education for any other student with special needs that preclude the student from participating in regular physical education instruction.
**PE professional development**	**PA17**	Staff qualifications and professional development	Professional development is provided to physical education teachers	**0**	Not addressed
				**1**	State law, regulation, or district policy addresses providing professional development to all PE teachers<br><br>**Example(s)**<br>• *PE staff will receive training on a yearly basis.*<br>• *The commissioner of education shall make prominently available on the department's website Internet sites that provide professional development for elementary teachers of physical education. These links must provide elementary teachers with information concerning current physical education and nutrition philosophy and best practices that result in student participation in physical activities that promote lifelong physical and mental well-being.*
**Physical Activity throughout the day**	**PA18**	Standards	Physical activity is provided throughout the school day and incorporated into the classroom (ex. classroom breaks)	**0**	Not addressed
				**1**	State law, regulation, or district policy recommends physical activity incorporated throughout the school day (ex. classroom breaks)<br><br>**Example**<br>• *The developmentally appropriate daily physical activity policy adopted by each local school board shall recommend practices which encourage the integration of health and physical activity across the curriculum.*
				**2**	State law, regulation, or district policy requires physical activity incorporated throughout the school day (ex. classroom breaks)<br><br>**Example(s)**<br><br>• *All students will have opportunities, encouragement, and support to engage in physical activity on a regular basis.*<br>• *Physical activity will be integrated into the curriculum where appropriate.*

Variable		Domain	Measure	Score	Brief Description
					• *Classrooms shall incorporate short breaks that include physical movement.*<br>• *Public schools shall seek to maximize physical activity by means including extending the school day, encouraging students to walk or bike to school, promoting active recess, including physical activity in after-school activities, supporting athletic programs, and integrating movement into classroom instruction.*
**Recess**	**PA19**	Program requirement	Addresses the frequency of recess	**0**	Not addressed
				**1**	State law, regulation, or district policy recommends recess<br><br>**Example**<br>• *Students will be given opportunities to be physically active throughout the day through activities such as recess, physical education, and after school athletics.*
				**2**	State law, regulation, or district policy requires recess less than daily<br><br>**Example**<br>• *Students will be given time for recess. (frequency is not stated)*
				**3**	State law, regulation, or district policy requires daily recess<br><br>**Example(s)**<br>• *All elementary school students will have 20 minutes of recess daily.*<br>• *Each school district board shall provide at least 100 minutes of supervised, safe, and unstructured free-play recess each week for students in kindergarten through grade 5 so that there are at least 20 consecutive minutes of recess per day.*
**Sports participation fees--allowed**	**PA21**	Program requirement	Allows fees for participation in school sports, sometimes referred to as "pay-to-play"	**0**	Not addressed
				**1**	State law, regulation, or district policy specifically allows for fees for participation<br><br>**Example(s)**<br>• *All students who participate in extracurricular activities must pay a $50 activity fee.*<br>• *A governing body may require and collect fees or other funds from or on behalf of students or require students to provide specialized equipment or specialized attire for any of the following purposes, including participation in*

Variable		Domain	Measure	Score	Brief Description
					extracurricular activities.
				2	State law, regulation, or district policy requires fees for participation, but provides for waivers or a cap on the amount of money to be collected.<br><br>**Example(s)**<br>• *All students who participate in extracurricular activities must pay a $50 activity fee. A sliding scale is available for those students who participate in the National School Lunch Program.*<br>• *Fees may be charged in connection with any school-sponsored activity which does not take place during the regular school day if the participation is voluntary and does not affect a student's grade or ability to participate fully in any course taught during the regular school day. An LEA shall provide for adequate waivers or other provisions in lieu of fee waivers to ensure that no student is denied the opportunity to participate in a class or school-sponsored or supported activity because of an inability to pay a fee.*
**Sports participation fees—prohibited**	**PA22**	Program requirement	Prohibits fees for participation in school sports, sometimes referred to as "pay-to-play"	**0**	Not addressed
				**1**	State law, regulation, or district policy prohibits fees for participation<br><br>**Example(s)**<br>• *There is no fee to participate in school sports.*<br>• *A pupil enrolled in a public school shall not be required to pay a pupil fee for participation in an educational activity. "Educational activity" means an activity offered by a school, school district, charter school, or county office of education that constitutes an integral fundamental part of elementary and secondary education, including, but not limited to, curricular and extracurricular activities.*

<table>
<tr><th colspan="2">Variable</th><th>Domain</th><th>Measure</th><th>Score</th><th>Brief Description</th></tr>
<tr><td rowspan="3">Breakfast at school</td><td rowspan="3">NS2</td><td rowspan="3">Policy requirement</td><td rowspan="3">Addresses <u>strategies to provide breakfast to students</u> (ex. SBP, grab and go, coordinating bus schedules, breakfast after the bell)</td><td>0</td><td>Not addressed</td></tr>
<tr><td>1</td><td>State law, regulation, or district policy recommends strategies to ensure students eat breakfast in the morning or maintains a program that only applies to certain schools (ex. schools with 70% FRPL students)

Example
• A public school operated by a school district of the State participating in the federal school breakfast program is encouraged to increase the number of students participating in the program by establishing a “breakfast after the bell” program through the incorporation of school breakfast in the first-period classroom or during the first few minutes of the school day.</td></tr>
<tr><td>2</td><td>State law, regulation, or district policy requires specific strategies to ensure all students eat breakfast

Example
• Beginning in school year 2017-18, every public school site, including charter school sites, participating in the community eligibility provision, shall be required to offer a breakfast at no cost to every student in the school through an alternative service model. “Alternative service model” means breakfast meal service that may include 1 or more of the following: 1) Breakfast in the Classroom; 2) Grab-and-Go Breakfast; 3) Second-Chance Breakfast.</td></tr>
<tr><td rowspan="3">Other food and beverages sold (competitive foods)—elementary school</td><td rowspan="3">NS3</td><td rowspan="3">Federal requirement</td><td rowspan="3">Addresses <u>nutrition standards for food and beverages sold outside of school meals</u> and the extent to which they must meet Smart Snacks in School in <u>elementary school</u></td><td>0</td><td>Not addressed</td></tr>
<tr><td>1</td><td>State law, regulation, or district policy recommends nutrition standards for food sold outside of school meal programs</td></tr>
<tr><td>2</td><td>State law, regulation, or district policy requires nutrition standards for food and beverages sold outside of school meals programs that do not meet Smart Snacks

Example
• All food and beverages sold will meet the Dietary Guidelines for Americans.
• Public schools, through a vending machine, school</td></tr>
</table>

Variable		Domain	Measure	Score	Brief Description
					cafeteria or school food service establishment during school hours, shall not make available to students enrolled in kindergarten through grade 12 any food or beverage containing industrially produced trans fat or use food containing industrially produced trans fat in the preparation of a food item served to those students. (no other standards are listed)
				**3**	State law, regulation, or district policy requires that food and beverages sold outside of school meal program meet federal Smart Snacks nutrition standards<br><br>**Example**<br>• *All foods and beverages, other than meals reimbursed under programs authorized by the Richard B. Russell National School Lunch Act and the Child Nutritional Act of 1966 (42 U.S.C. 1751 et seq.) that are available for sale to students a school during the school day must meet or exceed the nutrition requirements prescribed for such food and beverages by the United States Secretary of Agriculture under 7 CFR 210.11.*
**Other food and beverages sold (competitive foods)—middle school**	**NS4**	Federal requirement	Addresses <u>nutrition standards for food and beverages sold outside of school meals</u> and the extent to which they must meet Smart Snacks in School in <u>middle school</u>	**0**	Not addressed
				**1**	State law, regulation, or district policy recommends nutrition standards for food sold outside of school meal programs
				**2**	State law, regulation, or district policy requires nutrition standards for food sold outside of school meals programs that do not meet Smart Snacks<br><br>**Example(s)**<br>• *All food and beverages sold will meet the Dietary Guidelines for Americans.*<br>• *Public schools, through a vending machine, school cafeteria or school food service establishment during school hours, shall not make available to students enrolled in kindergarten through grade 12 any food or beverage containing industrially produced trans fat or use food containing industrially produced trans fat in the preparation of a food item served to those students. (no other standards are listed)*
				**3**	State law, regulation, or district policy requires that food sold outside of school meal program meet federal Smart Snacks nutrition standards<br><br>**Example**

Variable		Domain	Measure	Score	Brief Description
					• *All foods and beverages, other than meals reimbursed under programs authorized by the Richard B. Russell National School Lunch Act and the Child Nutritional Act of 1966 (42 U.S.C. 1751 et seq.) that are available for sale to students a school during the school day must meet or exceed the nutrition requirements prescribed for such food and beverages by the United States Secretary of Agriculture under 7 CFR 210.11.*
**Other food and beverages sold (competitive foods)—high school**	**NS5**	Federal requirement	Addresses nutrition standards for food and beverages sold outside of school meals and the extent to which they must meet Smart Snacks in School in high school	**0**	Not addressed
				**1**	State law, regulation, or district policy recommends nutrition standards for food sold outside of school meal programs
				**2**	State law, regulation, or district policy requires nutrition standards for food sold outside of school meals programs that do not meet Smart Snacks<br><br>**Example(s)**<br>• *All food and beverages sold will meet the Dietary Guidelines for Americans.*<br>• *Public schools, through a vending machine, school cafeteria or school food service establishment during school hours, shall not make available to students enrolled in kindergarten through grade 12 any food or beverage containing industrially produced trans fat or use food containing industrially produced trans fat in the preparation of a food item served to those students. (no other standards are listed)*
				**3**	State law, regulation, or district policy requires that food sold outside of school meal program meet federal Smart Snacks nutrition standards<br><br>**Example**<br>• *All foods and beverages, other than meals reimbursed under programs authorized by the Richard B. Russell National School Lunch Act and the Child Nutritional Act of 1966 (42 U.S.C. 1751 et seq.) that are available for sale to students a school during the school day must meet or exceed the nutrition requirements prescribed for such food and beverages by the United States Secretary of Agriculture under 7 CFR 210.11.*
**Class parties in**	**NS6**	Standards	Addresses nutrition standards for classroom	**0**	Not addressed

Variable		Domain	Measure	Score	Brief Description
**elementary school**			parties in elementary school	**1**	State law, regulation, or district policy addresses nutrition standards for class parties<br><br>**Example(s)**<br>• *Classroom parties should provide healthy alternatives such as fruits and vegetables.*<br>• *Each school board is encouraged to adopt policies ensuring that every student has access to healthful items for fundraisers, classroom parties, and rewards in the schools.*
				**2**	Requires nutrition standards for class parties<br><br>**Example(s)**<br>• *Foods and beverages provided for classroom parties will be limited to water, 100% juice, fruits and vegetables.*<br>• *Foods and beverages provided for class parties must meet the district nutrition standards. (and standards are clearly established)*
**Free potable water during the school day**	**NS8**	Program requirement	Addresses providing free, potable drinking water to students throughout the school campus, during the school day	**0**	Not addressed
				**1**	State law, regulation, or district policy recommends making free potable water available at all times during the school day<br><br>**Example**<br>• *Each school board is encouraged to adopt policies ensuring that every student has access to a school facility with a sufficient number of functioning water fountains or other means which provide him or her with sufficient water.*
				**2**	State law, regulation, or district policy requires making free potable water available at all times during the school day<br><br>**Example**<br>• *Public schools shall, in accordance with applicable guidelines of the Department, make readily available plain, potable water to all students during the school day, at no cost to the students.*
**Unpaid meal charge policy--supports students**	**NS10a**	Program requirement	Establishes a policy regarding unpaid meal charges that prohibits shaming and/or denying meals	**0**	Not addressed
				**1**	State law, regulation, or district policy establishes a policy regarding unpaid meal charges that prohibits shaming and/or denying meals<br><br>**Example(s)**<br>• *A local educational agency shall ensure that a pupil whose parent or guardian has unpaid school meal fees is not*

Variable		Domain	Measure	Score	Brief Description
					shamed, treated differently, or served a meal that differs from what a pupil whose parent or guardian does not have unpaid school meal fees would receive under that local educational agency's policy.<br>• *A local educational agency shall not take any action directed at a pupil to collect unpaid school meal fees. A local educational agency may attempt to collect unpaid school meal fees from a parent or guardian, but shall not use a debt collector.*<br>• *A local educational agency shall notify a parent or guardian of the negative balance of a pupil's school meal account no later than 10 days after the pupil's school meal account has reached a negative balance. Before sending this notification, the local educational agency shall exhaust all options and methods to directly certify the pupil for free or reduced-price meals.*
**Unpaid meal charge policy—restricts access**	**NS10b**	Program requirement	Establishes a policy regarding unpaid meal charges that allows schools to overtly identify students and/or deny meals	**0**	Not addressed
				**1**	State law, regulation, or district policy establishes a policy regarding unpaid meals charges that allows schools to overtly identify students and/or deny meals.<br><br>**Example**<br>• *In the event that a school district determines that a student's school breakfast or school lunch bill is in arrears, the district shall contact the student's parent or guardian to provide notice of the arrearage and shall provide the parent or guardian with a period of 10 school days to pay the amount due. If the student's parent or guardian has not made full payment by the end of the 10 school days, then the district shall again contact the student's parent or guardian to provide notice that school breakfast or school lunch, as applicable, shall not be served to the student beginning one week from the date of the second notice unless payment is made in full.*
**Restricts marketing of unhealthy choices**	**NS12**	Federal requirement	Addresses restricting the marketing of unhealthy choices that cannot be sold during the school day (i.e. those that do not meet Smart Snacks)	**0**	Not addressed
				**1**	State law, regulation, or district policy recommends restricting marketing of foods and beverages to those that meet Smart Snacks<br><br>**Example(s)**<br>• *Marketing in schools should avoid unhealthy products.*<br>• *School-based marketing will be limited to the promotion of healthy items (unclear what constitutes a "healthy" item).*

Variable		Domain	Measure	Score	Brief Description
					• *School-based marketing will be consistent with the district nutrition standards (nutrition standards do not meet Smart Snacks).*
				**2**	State law, regulation, or district policy requires restricting advertising/marketing of foods and beverages to those that meet Smart Snacks<br><br>**Example(s)**<br>• *School-based marketing will be consistent with items that meet Smart Snacks.*<br>• *School-based marketing will be consistent with the district nutrition standards (nutrition standards meet Smart Snacks).*

Health Services

Variable		Domain	Measure	Score	Brief Description
**School-based health services**	**HS1**	Policy requirement	Addresses the availability of school-based health services within the school district (including grant-based programs)	**0**	Not addressed
				**1**	State law, regulation, or district policy addresses aspects of traditional school-based health services (noted below), but does not formalize a program to aid in the establishment of clinics or health centers on campus for students. Health services provided for may:<br><br>• Establish a school health board to govern school district health services<br>• Set qualifications and certification required for physicians and health professionals providing school-based health services<br>• Provide financial support for school-based health services<br>• Address community involvement and/or partnership with school-based health services<br>• Provide details as to the type of services provided<br><br>**Example(s)**<br>• *School health services and school nurse services means health services that are designed to enable a child with a disability to receive FAPE as described in the child's IEP. School nurse services are services provided by a qualified school nurse. School health services are services that may be provided by either a qualified school nurse or other qualified person.*<br>*' All schools support and assist students in being healthy learners through promoting annual well child examinations, biannual dental examinations by a licensed dentist, up-to-date immunizations, emergency information, preventive health care and enrollment for children and families into health care insurance. A public/private partnership is easily accomplished through school nursing services, local WVDHHR/Bureau services, school-based services (including medical, mental and oral health), non-profit services, private sector services, businesses, etc. The school nurse is the school health expert who is qualified to lead the coordination and*

**Variable**		Domain	Measure	**Score**	Brief Description
					monitoring of health promotion through school screenings and examinations
				**2**	State law, regulation, or district policy addresses the development of school-based health centers or clinics, even if only through a grant program on some campuses.<br><br>**Example(s)**<br>• *The school-based health center (SBHC) program supports communities in promoting the health and well-being of the school-age population through the evidence-based best practice within a public health framework. These rules (OAR 333-028-0200 through 333-028-0250) establish the procedure and criteria the Oregon Health Authority shall use to certify, suspend and decertify SBHCs. Certification of a SBHC by the SBHC state program is voluntary; an operating clinic is free to choose not to participate in certification and still operate. Only certified SBHCs are eligible for funding from Oregon Health Authority.*<br>• *Subject to available funding, the State Department of Education, in consultation with the State Department of Health, shall establish the school health grant pilot program to improve student health by assisting local school districts in implementing a school health program. In order to qualify for a school health grant, a school district shall submit a detailed implementation plan, developed in accordance with the guidelines for a school health program developed by the State Department of Education, and including the following components:(a) A dedicated school health coordinator and technical and administrative support for collection of data and program evaluation.(b) A description of how the school district currently addresses physical activity, nutrition, and other obesity prevention measures.(c) A description of how the school district would use the state grant to augment what it is currently doing, including defining priorities based on the students' health need and meeting education performance indicators, developing an action plan for addressing those needs based on realistic goals and measurable objectives, establishing a timeline for implementation, and developing and maintaining a system to evaluate progress and outcomes for the program.(d) All school districts receiving grants will report annually to the State Department of Education progress*

Variable		Domain	Measure	Score	Brief Description
					towards the achievement of state education performance indicators and standards and requirements relating to physical activity and nutrition.
**Preventive health screenings required for at least 1 grade in elementary school**	**HS2a**	Standards	Addresses vision screening during elementary school	**0**	Not addressed
				**1**	State law, regulation, or district policy addresses vision screening<br><br>**Example**<br>• *Each child shall be screened periodically during elementary school for vision and hearing deficiencies by designated school personnel.*
	**HS2b**		Addresses hearing screening during elementary school	**0**	Not addressed
				**1**	State law, regulation, or district policy addresses hearing screening<br><br>**Example**<br>• *Each child shall be screened periodically during elementary school for vision and hearing deficiencies by designated school personnel.*
	**HS2c**		Addresses dental screening during elementary school	**0**	Not addressed
				**1**	State law, regulation, or district policy addresses dental screening<br><br>**Example**<br>• *A dental examination shall be provided for a student upon entry into school and in grades 3 and 7.*
**Preventive health screenings required for at least 1 grade in middle school**	**HS3a**	Standards	Addresses vision screening during middle school	**0**	Not addressed
				**1**	State law, regulation, or district policy addresses vision screening<br><br>**Example**<br>• *Each child shall be screened periodically during middle school for vision and hearing deficiencies by designated school personnel.*
	**HS3b**		Addresses hearing screening during middle school	**0**	Not addressed
				**1**	State law, regulation, or district policy addresses hearing screening<br><br>**Example**<br>• *Each child shall be screened periodically during middle*

Variable		Domain	Measure	Score	Brief Description
					school for vision and hearing deficiencies by designated school personnel.
	**HS3c**		Addresses dental screening during middle school	**0**	Not addressed
				**1**	State law, regulation, or district policy addresses dental screening<br><br>**Example**<br>• *A dental examination shall be provided for a student upon entry into school and in grades 3 and 7.*
**Preventive health screenings required for at least 1 grade in high school**	**HS4a**	Standards	Addresses vision screening during high school	**0**	Not addressed
				**1**	State law, regulation, or district policy addresses vision screening<br><br>**Example**<br>• *Each child shall be screened periodically during high school for vision and hearing deficiencies by designated school personnel.*
	**HS4b**		Addresses hearing screening during high school	**0**	Not addressed
				**1**	State law, regulation, or district policy addresses hearing screening<br><br>**Example**<br>• *Each child shall be screened periodically during high school for vision and hearing deficiencies by designated school personnel.*
	**HS4c**		Addresses dental screening during high school	**0**	Not addressed
				**1**	State law, regulation, or district policy addresses dental screening<br><br>**Example**<br>• *A dental examination shall be provided for a student upon entry into high school.*
**Chronic Condition: Plans for specific chronic conditions**	**HS7a**	Policy requirement	Asthma plan developed as a stand-alone policy or as part of larger plan to manage chronic health conditions (even if only in regards to self-administration of asthma medication)	**0**	Not addressed
				**1**	State law, regulation, or district policy addresses asthma plan<br><br>**Example**<br>• *The department of health shall develop a comprehensive state plan to reduce the burden of asthma on school children in this state. The comprehensive plan shall, at a*

Variable		Domain	Measure	Score	Brief Description
					minimum, promote the development of school asthma action plans between LEAs and local health agencies. School asthma action plans shall include emergency protocols for medical emergencies due to asthma complications. The comprehensive plan shall encourage schools to have individual asthma action plans for students with asthma.
	**HS7b**		Diabetes plan developed as stand-alone policy or as part of a larger plan to manage chronic health conditions (even if only in regards to self-administration of glucose)	**0**	Not addressed
				**1**	State law, regulation, or district policy addresses diabetes plan<br><br>**Example**<br>• *The school district government board may adopt policies and procedures for pupils who have been diagnosed with diabetes by a health professional to manage their diabetes in the classroom, on school grounds, and at school sponsored activities as authorized by their primary health professional.*
	**HS7c**		Allergy plan developed as stand-alone policy or as part of a larger plan to manage chronic health conditions (even if only in regards to self-administration of epinephrine)	**0**	Not addressed
				**1**	State law, regulation, or district policy addresses allergy plan<br><br>**Example**<br>• *Each school district shall keep on file a plan for the emergency administration of anaphylaxis medication. Student allergies shall be well documented. The school district will designate the employee responsible for obtaining the Epinephrine injectors at each school site.*
**Referrals**	**HS8**	Program requirement	Addresses providing follow-up with parents to recommend referrals to private physicians or community health partners as part of follow-up to health conditions or preventive health screenings	**0**	Not addressed
				**1**	State law, regulation, or district policy addresses providing referrals to private physicians or community health partners<br><br>**Example(s)**<br>• *In the event that a student fails the yearly vision test, the school nurse will provide a referral to [Local Health Clinic] to receive a full eye exam.*<br>• *Following the health assessment, school districts shall demonstrate a capability for referral to appropriate medical facilities for follow up care when necessary.*

Variable		Domain	Measure	Score	Brief Description
**School nurse availability**	**HS10**	Standard	Establishes availability of school nurse in school buildings across campus and at all grade levels	**0**	Not addressed
				**1**	State law, regulation, or district policy addresses school nurses in one of the following ways:<br>• At least 1 nurse will be available throughout the district, but not in every school<br>• At least 1 nurse will be available, but not across all grades<br>• School nurses are available on campus, but not every day or not at all times during the school day<br>• School district is required to employ a school nurse, but not each individual school<br><br>**Example(s)**<br>• *A school nurse will be on campus in all high schools throughout the district (and there is no mention of school nurses in elementary or middle schools).*<br>• *Each county board shall employ full time at least one school nurse for every one thousand five hundred kindergarten through seventh grade pupils in net enrollment or major fraction thereof.* (not clear that a nurse will be present on each school campus)
				**2**	State law, regulation, or district policy requires full-time school nurses in every school, every day during which students are present.<br><br>**Example**<br>• *At least one school nurse shall be available at each school campus during the school day while students are present.*
**School nurse professional development**	**HS12**	Staff qualifications and professional development	Professional development provided to all school nurses	**0**	Not addressed
				**1**	State law, regulation, or district policy addresses providing professional development to all school nurses<br><br>**Example(s)**<br>• *School nurses will receive 5 hours of additional training per year to assist in the recognition of mental health issues in students.*<br>• *The school nurse shall comply with professional nursing practice standards, including professional development related to licensure.*

Employee Wellness

Variable		Domain	Measure	Score	Brief Description
**Staff wellness program**	**EW1**	Program requirement	Addresses the establishment of a <u>wellness program for school staff</u>	**0**	Not addressed
				**1**	State law, regulation, or district policy recommends wellness program for school staff<br><br>**Example(s)**<br>• *The local school health advisory council's duties include recommending policies, procedures, strategies, and curriculum for school employee wellness.*<br>• *Schools should provide at least three wellness activities per school year.*
				**2**	State law, regulation, or district policy requires wellness program for school staff<br><br>**Example(s)**<br>• *Each LEA shall develop and implement policies and protocols that promote the health of school employees to support their overall well-being and their performance as educators and role models, including, as necessary, wellness programs, employee assistance programs, referral systems, and/or other services or supports as may be needed to help school staff maintain healthy lifestyles.*<br>• *School district wellness policies shall include a plan addressing the staff wellness needs of all school staff that minimally ensures an equitable work environment and meets the American with Disabilities Act.*
	**EW1b**		<u>Stress management</u> addressed as stand-alone policy or as part of staff wellness program	**0**	Not addressed
				**1**	State law, regulation, or district policy addresses stress management<br><br>**Example**<br>• *Schools can provide the following activities to promote staff wellness: health screenings (height, weight, cholesterol, blood pressure); assistance programs (stress management, smoking cessation); fitness activities (running clubs, yoga); and nutrition programs (weight loss programs).*
	**EW1d**		<u>Health assessment</u> addressed as stand-alone policy or as part of staff wellness program	**0**	Not addressed or only as a condition of employment (e.g. drug testing)

Variable		Domain	Measure	Score	Brief Description
				**1**	State law, regulation, or district policy addresses health assessment, for the purpose of preventive health of the employee<br><br>**Example**<br>• *Schools can provide the following activities to promote staff wellness: health screenings (height, weight, cholesterol, blood pressure); assistance programs (stress management, smoking cessation); fitness activities (running clubs, yoga); and nutrition programs (weight loss programs).*
**Staff physical activity**	**EW2**	Standard	Addresses providing physical activity opportunities for school staff through school activities or partnerships with PA programs	**0**	Not addressed
				**1**	State law, regulation, or district policy addresses providing physical activity opportunities for school staff<br><br>**Example(s)**<br>• *Staff will have access to physical activity opportunities, including the school fitness center.*<br>• *All strategic plans shall include strategies to decrease obesity and improve the health and wellness of students and employees through nutrition, physical activity, health education, and physical education.*
**Healthy food policies for school staff**	**EW3**	Standard	Addresses providing healthy food for staff on campus, during meetings, in vending machines, etc.	**0**	Not addressed
				**1**	State law, regulation, or district policy addresses providing healthy food for staff<br><br>**Example(s)**<br>• *Staff meetings will provide healthy choices, such as fruits and vegetables.*<br>• *All staff events must meet the healthy food policy developed by the staff wellness committee.*

Family Engagement

Variable		Domain	Measure	Score	Brief Description
**Family engagement - plans or policies**	**FE2**	Policy or Plan Requirement	Directs local education agencies (LEAs) to develop or implement plans, policies, or programs to engage parents/families in the educational process.	**0**	Not addressed
				**1**	State law, regulation, or district policy encourages districts to adopt plans, policies, or strategies to engage parents and families in the educational process.<br>**Example**<br>• *The State Board of Education shall develop a program to encourage local and regional boards of education to develop and implement plans to involve parents of students in the educational process in that district and to increase community involvement in the schools.*
				**2**	State law, regulation, or district policy requires districts to adopt plans, policies, or strategies to engage parents and families in the educational process.<br>**Example**<br>• *Each school district board of education shall adopt a district policy for increasing and supporting parent engagement in the public schools.*
**Family engagement – professional development**	**FE3**	Staff Qualifications and Professional Development	Establishes professional development, training, or education programs for teachers, administrators, and other school staff on increasing parent/family engagement.	**0**	Not addressed
				**1**	State law, regulation, or district policy encourages districts to provide professional development for school personnel on parent and family engagement.<br>**Example**<br>• *As part of the district parent engagement policy, a district is encouraged to provide training concerning best practices and skills for district and school personnel in working with parents.*
				**2**	State law, regulation, or district policy requires districts to provide professional development for school personnel on parent and family engagement.

Variable		Domain	Measure	Score	Brief Description
					**Example**<br>• *(2) Two (2) hours of professional development as part of the minimum number of hours of professional development required for administrators, designed to enhance understanding of:(A) Effective parent involvement strategies; and (B) The importance of administrative leadership in setting expectations and creating a climate conducive to parental participation.*
**Family engagement – local governance**	**FE4**	Stakeholders	Establishes local governance councils, advisory boards, or committees that encourage or require parent/family engagement in school decision-making.	**0**	Not addressed
				**1**	State law, regulation, or district policy encourages parent or family member representation on school governance councils or in school improvement planning efforts.<br>**Example**<br>*The Legislative Assembly recognizes that students in public elementary and secondary schools can best reach the levels of performance expected under the provisions of this chapter with parental and community participation in the education process. It is, therefore, recommended but not required that:*<br>*(1) School districts provide opportunities for parents or guardians to be involved in establishing and implementing educational goals and to participate in decision-making at the school site;*
				**2**	State law, regulation, or district policy requires parent or family member representation on school governance councils or in school improvement planning efforts.<br>**Example**<br>*A. Each public school shall create an advisory "school council" to assist the school principal with school-based decision-making and to involve parents in their children's education.*<br>*B. A school council shall be created and its membership elected in accordance with local school board rule. School council membership shall reflect an equitable balance between school employees and parents and community members. At least one community member shall represent the business community, if such person is available. The school principal may serve as chairman. The school principal shall be an active member of the school council.*

Variable		Domain	Measure	Score	Brief Description
**Family engagement – school discipline**	**FE5**	Stakeholders	Integrates family and parent input into policymaking around student discipline and behavior management (i.e., developing codes of conduct)	**0**	Not addressed
				**1**	State law, regulation, or district policy encourages parent or family member involvement in the development of student codes of conduct.<br><br>**Example**<br><br>• (*c) Local boards of education shall provide opportunities for parental involvement in developing and updating student codes of conduct.*
				**2**	State law, regulation, or district policy requires parent or family member involvement in the development of student codes of conduct.<br><br>**Example**<br><br>• *(a) Each governing body shall adopt a written school disciplinary and safety program… standards required under this paragraph must be developed and periodically reviewed with the collaboration of members of each school, parents, legal guardians, teachers, and other persons responsible for the students at a school;*

Community Involvement

Variable		Domain	Measure	Score	Brief Description
**Community involvement – local governance**	**CI1**	Stakeholders	Establishes local governance councils, advisory boards, or committees that encourage or require community engagement in school decision-making.	**0**	Not addressed
				**1**	State law, regulation, or district policy encourages community member representation on school governance councils or in school improvement planning efforts.<br><br>**Example**<br><br>*(d) A public school or district that receives a low performance designation under this section shall prepare and submit to the department a school or district improvement plan, as applicable, in accordance with regulations adopted by the board. The improvement plan must be prepared with the maximum feasible public participation of the community including, as appropriate, interested individuals, teachers, parents, parent organizations, students, tribal organizations, local government representatives, and other community groups.*
				**2**	State law, regulation, or district policy requires community member representation on school governance councils or in school improvement planning efforts.<br><br>**Example**<br><br>*(a) The district school board shall establish an advisory council for each school in the district and shall develop procedures for the election and appointment of advisory council members. Each school advisory council shall include in its name the words "school advisory council." The school advisory council shall be the sole body responsible for final decision making at the school relating to implementation of ss. 1001.42(18) and 1008.345. A majority of the members of each school advisory council must be persons who are not employed by the school district. Each advisory council shall be composed of the principal and an appropriately balanced number of teachers, education support employees, students, parents, and other business and community citizens who are representative of the ethnic, racial, and economic community served by the school.*

Variable		Domain	Measure	Score	Brief Description
**Community involvement – school community partnerships**	**CI3**	Partnerships	Encourages or requires creation of interagency agreements or school-community partnerships to address student and family needs (includes mental health and juvenile justice partnerships).	**0**	Not addressed
				**1**	State law, regulations, or policy encourages districts to establish school-community partnerships to address student needs.<br><br>**Example**<br>*County boards of education are encouraged to establish county agency and organization partnerships with the purpose of providing the county's schools with additional supports and resources to shape behaviors in safe and supportive schools. These partnerships may be both formal and informal as described under Section 2***.**
				**2**	State law, regulations, or policy requires districts to establish school-community partnerships to address student needs.<br><br>**Example**<br>*Superintendents and principals in every school district in the commonwealth shall pursue opportunities to establish school–community partnerships that may advance policy development, staff development, curriculum development, instructional enrichment and may provide material and financial support. The commissioner of education shall assist in and facilitate with the establishment of school–community partnerships. Subject to appropriation, the board shall establish a grant program to assist school districts in developing and implementing such partnerships.*
**Community use of public school facilities**	**CI4**	Partnerships	Addresses community use of school facilities.<br><br>**Aligns with NASBE State School Health Policies Database, Health Promoting Environment, Shared Use Agreements	**0**	Not addressed
				**1**	State law, regulations, or district policy authorizes the community use of school buildings and property for recreation or other purposes.<br><br>**Example**<br>*(30) May make available school facilities and equipment for specified public purposes if such purposes appear, in the judgment of the board, to be in the best interests of the district and are an efficient, economical, and appropriate use of the facilities*

**Variable**		Domain	Measure	Score	Brief Description
					and equipment.
				**2**	State law, regulations, or district policy encourages and/or incentivizes the community use of school buildings and property for recreation or other purposes.<br><br>**Example**<br>*All public school buildings, facilities, and grounds shall be available for general recreational purposes, and for public and community use, whenever these activities do not interfere with the normal and usual activities of the school and its pupils… The department may assess and collect fees and charges from the users of school buildings, facilities, grounds, and equipment, which include fees and charges assessed and collected by the department for parking on roadways and in parking areas under the jurisdiction of the department,…*

Variable		Domain	Measure	Score	Brief Description
**Trauma-informed practices**	**CPS2**	Staff Qualifications and Professional Development	Incorporates instruction on trauma-informed practices into professional development and teacher education programs.	**0**	Not addressed
				**1**	State law, regulation, or district policy encourages districts to provide professional development for school personnel on trauma-informed practices.<br><br>**Example**<br>*The State Board of Education, within available appropriations and utilizing available materials, shall assist and encourage local and regional boards of education to include: (I) trauma-informed practices for the school setting to enable teachers, administrators and pupil personnel to more adequately respond to students with mental, emotional or behavioral health needs;*
				**2**	State law, regulation, or district policy requires districts to provide professional development for school personnel on trauma-informed practices.<br><br>**Example**<br>*The state superintendent shall…(63) Mental health training program. Establish a mental health training support program under which the department provides training on all of the following evidence-based strategies related to addressing mental health issues in schools to school district staff and instructional staff of charter schools under s. 118.40 (2r) or (2x):*<br>*(a) The screening, brief intervention, and referral to treatment program.*<br>*(b) Trauma sensitive schools.*<br>*(c) Youth mental health first aid.*
**Mental health professional development**	**CPS3**	Staff Qualifications and Professional Development	Incorporates instruction on recognizing signs and symptoms of mental health issues (e.g., Mental Health First Aid) into professional development and teacher education programs.	**0**	Not addressed
				**1**	State law, regulation, or district policy encourages districts to provide professional development for school personnel on youth mental health.

Variable		Domain	Measure	Score	Brief Description
					**Example(s)**<br>• *Subject to appropriation for this specific purpose, the department shall provide funds for mental health first aid training targeted at teachers and educational staff.*<br>• *The division shall make the mental health first aid training program available to licensed teachers, school counselors,…" OR encourages participation in mental health first aid training by allowing educators continuing education credits.*
				**2**	State law, regulation, or district policy requires districts to provide professional development for school personnel on youth mental health.<br><br>**Example(s)**<br>• *The state superintendent shall…(63) Mental health training program. Establish a mental health training support program under which the department provides training on all of the following evidence-based strategies related to addressing mental health issues in schools to school district staff and instructional staff of charter schools under s. 118.40 (2r) or (2x):*<br>*(a) The screening, brief intervention, and referral to treatment program.*<br>*(b) Trauma sensitive schools.*<br>*(c) Youth mental health first aid.*<br>• *Mental health first aid training program. Each such district safe school climate coordinator shall successfully complete such mental health first aid training. (d) Each local and regional board of education may require teachers, school nurses, counselors and other school employees to participate in mental health first aid training.*
**Early identification and referral for mental health needs**	**CPS4**	Programs and Services	Encourages or requires districts to establish early identification and referral processes and networks to link students with mental, behavioral health, or social services needs to intervention services.<br><br>**Aligns with NASBE State School Health Policies Database, Student Services,	**0**	Not addressed
				**1**	State law, regulation, or district policy authorizes districts to conduct mental health screening in schools or to disseminate information on mental health resources.<br><br>**Example(s)**

**Variable**		Domain	Measure	Score	Brief Description
			Counseling and Mental Health Services		• *(b) Universal mental health or socioemotional screening is only permitted under the following circumstances:(1) A parent, guardian, legal custodian or caregiver …has provided written, active, informed and voluntarily signed consent…*<br>• *(a) Notwithstanding AS 14.30.171(a)(4), a school district may make available to an interested parent or guardian a list of community resources, including mental health services if the list conspicuously states the following: "This list is provided as a resource to you. The school neither recommends nor requires that you use this list or any of the services provided by individuals or entities on the list. It is for you to decide what services, if any, to use and from whom you wish to obtain them.*
				**2**	State law, regulation, or district policy encourages or requires districts to implement identification and referral processes to link students and families with needed resources.<br><br>**Example**<br>*(a) In each fiscal year for which funds are appropriated for purposes of the primary mental health program, the department shall establish a grant program for the purpose of providing funds to local and regional boards of education for the establishment of school-based programs for the detection and prevention of emotional, behavioral and learning problems in public school children primarily in grades kindergarten through grade three.*
**Suicide prevention - professional development**	**CPS5**	Staff Qualifications and Professional Development	Incorporates instruction in suicide prevention into professional development and teacher education programs.	**0**	Not addressed
				**1**	State law, regulation, or district policy encourages districts to provide professional development for school personnel on youth suicide prevention<br><br>**Example**<br>*The board of a school district or board of directors of a public school academy is encouraged to provide age-appropriate instruction for pupils and professional development for school personnel concerning the warning signs and risk factors for suicide and depression and the protective factors that help*

Variable		Domain	Measure	Score	Brief Description
					prevent suicide.
				2	State law, regulation, or district policy requires districts to provide professional development for school personnel on youth suicide prevention.<br><br>**Example(s)**<br>• *A school district and the department shall provide youth suicide awareness and prevention training approved by the commissioner to each teacher, administrator, counselor, and specialist who is employed by the district or department to provide services to students in a public school in the state at no cost to the teacher, administrator, counselor, or specialist.*<br>• *Each school district and charter school shall require its employees to receive 12.5 hours of training every 3 years consisting of all of the following: Four and one-half hours of a suicide prevention training program established under § 4165(a) of this title, with each school district and charter school employee receiving 90 minutes of such training each year.*
**Suicide prevention policy**	**CPS6**	Policy or Plan Requirements	Encourages or requires districts to <u>adopt local suicide prevention policies.</u><br><br>**Aligns with NASBE State School Health Policies Database, Student Services, Counseling and Mental Health Services	**0**	Not addressed
				**1**	State law, regulation, or district policy encourages districts to adopt suicide prevention policies.<br><br>**Example**<br>*The board of education of each school district in this state may adopt a policy regarding suicide awareness and training and the reporting of student drug abuse.*
				**2**	State law, regulation, or district policy requires districts to adopt suicide prevention policies.<br><br>**Example**<br>*Each LEA shall adopt a policy on student suicide prevention. The policies shall be developed in consultation with school and community stakeholders, school-employed mental health professionals, and suicide prevention experts, and shall, at a*

Variable		Domain	Measure	Score	Brief Description
					minimum, address procedures relating to suicide prevention, intervention, and postvention.
**School counseling services – grades K-8**	**CPS7**	Programs and Services	Encourages or requires the provision of school counseling services in elementary grade levels (K-8).	**0**	Not addressed
				1	State law, regulation, or district policy encourages the provision of school counseling or guidance services in elementary grade levels (K-8).<br><br>**Example**<br>*The governing board of a school district may provide a comprehensive educational counseling program for all pupils enrolled in the school district. It is the intent of the Legislature that a school district that provides educational counseling to its pupils implement a structured and coherent counseling program.*
				**2**	State law, regulation, or district policy requires the provision of school counseling or guidance services in elementary grade levels (K-8).<br><br>**Example**<br>*Each parish and city school board shall provide elementary school guidance counselors for all students in kindergarten through grade six in the public elementary schools in the state… Such guidelines shall require that there shall be one guidance counselor for every four hundred students.*
**School counseling services – grades 9 -12**	**CPS8**	Programs and Services	Encourages or requires the provision of school counseling services in secondary grade levels (9-12).	**0**	Not addressed
				**1**	State law, regulation, or district policy encourages the provision of school counseling or guidance services in secondary grade levels (9-12).<br><br>**Example**<br>*(1) Each district school board shall annually submit a district guidance report to the Commissioner of Education by June 30.*<br>*(2) The guidance report shall include, but not be limited to, the following:*<br>*(a) Examination of student access to certified school*

Variable		Domain	Measure	Score	Brief Description
					counselors.<br>*(b) Degree to which a district has adopted or implemented a guidance model program.*<br>*(c) Evaluation of the information and training available to certified school counselors and career specialists to advise students on areas of critical need, labor market trends, and technical training requirements.*<br>*(d) Progress toward incorporation of best practices for advisement as identified by the department.*<br>*(e) Consideration of alternative guidance systems or ideas, including, but not limited to, a teacher-advisor model, mentoring, partnerships with the business community, web-based delivery, and parental involvement.*<br>*(f) A guidance plan for the district.*
				**2**	State law, regulation, or district policy requires the provision of school counseling or guidance services in secondary grade levels (9-12).<br><br>**Example(s)**<br><br>*(a) District Comprehensive Guidance and Counseling. Each school district shall provide a coordinated comprehensive guidance and counseling program to support the academic, career, personal/social, and community involvement development of each and every student.*<br><br>*(3) Guidance Staff Assignments. Each school district shall maintain a licensed staff and promote effective guidance practices consistent with the district's expected comprehensive guidance and counseling program outcomes. In determining staffing for the program, the following shall be considered:(a) Alignment with the American School Counselor Association recommended student to counselor ratio of 250:1;*<br>*(b) The number of aides or clerical staff assigned to support the implementation of the comprehensive guidance and counseling program.*
**School-based mental health programs**	**CPS10**	Programs and Services	Establishes <u>school-based or school-linked mental health promotion and intervention programs or designates student support staff such as school social workers.</u>	**0**	Not addressed
				**1**	State law, regulation, or district policy encourages districts to establish school-based or school-linked mental health promotion and intervention programs.

Variable		Domain	Measure	Score	Brief Description
			**Aligns with NASBE State School Health Policies Database, Student Services, Counseling and Mental Health Services		**Example(s)**<br>• *Before July 1, 2016, each school corporation may enter into a memorandum of understanding with a community mental health center established under IC 12-29-2 or a provider certified or licensed by the division of mental health and addiction to establish conditions or terms for referring students of the school corporation to the mental health care provider or community mental health center for services.*<br>• *(a) In each fiscal year for which funds are appropriated for purposes of the primary mental health program, the department shall establish a grant program for the purpose of providing funds to local and regional boards of education for the establishment of school-based programs for the detection and prevention of emotional, behavioral and learning problems in public school children primarily in grades kindergarten through grade three.*
				**2**	State law, regulation, or district policy requires districts to establish school-based or school-linked mental health promotion and intervention programs.<br>**Example**<br>*G-14-3.2. Psychological and Mental Health Services. Mental and emotional health issues directly impede students' abilities to learn. Such issues include bullying, alcohol and drug abuse, depression, anxiety, and domestic violence, as well as psychiatric disorders. Therefore, each LEA shall:*<br>*-- Ensure that students have access to a coordinated program of culturally and linguistically responsive psychological and mental health services, on site or through effective referral systems;*<br>*-- Ensure that school psychological and mental health services will be provided by appropriately credentialed, high quality staff. Services must provide for identification of risks and assessment of service needs; primary prevention; individual, family, and group counseling; consultative services; and resource and service coordination; and*<br>*-- To the extent practicable, ensure that schools coordinate with community youth development, prevention, and treatment efforts.*

Variable		Domain	Measure	Score	Brief Description
**School-wide positive behavioral supports**	**SE2**	Programs and Services (S/D)	Addresses use of positive behavioral supports with tiered interventions to communicate and reinforce behavioral expectations and provide selective and intensive interventions.	**0**	Not addressed
				**1**	State law, regulation, or district policy encourages districts to implement school-wide positive behavioral interventions or tiered frameworks.<br><br>**Example(s)**<br>• *(b) Program established -- Suspension. -- (1) Subject to paragraph (3) of this subsection, each county board shall require an elementary school that has a suspension rate that exceeds the standard specified in paragraph (2) of this subsection to implement: (i) A positive behavioral interventions and support program;*<br>• *Local boards of education are encouraged to implement PBIS and RTI programs and initiatives in their schools, and particularly in high need schools.*<br>• *Rule 1. A school social worker may function in any of the following roles:*<br>○ *Provide individual and group counseling to students and their families in need of assistance utilizing an ecological framework addressing variations in development and learning, as well as reciprocal influences*<br>○ *of home, school, and community.*<br>○ *Provide consultation, collaboration, and advisement services to students, their family members and school staff regarding students' social, emotional, and behavioral status impacting learning, development,*<br>○ *mental health, and school success. Encourage developmentally appropriate student self-determination and self-advocacy.*<br>○ *(c) Implement school social work services within a multi-tiered intervention model for programs and services.*

Variable		Domain	Measure	Score	Brief Description
				**2**	State law, regulation, or district policy requires districts to implement school-wide positive behavioral supports or tiered frameworks.<br><br>**Example(s)**<br><br>• *Positive Behavioral Supports and Discipline. Each LEA shall ensure that schools promote a positive climate with emphasis on mutual respect, self-control, good attendance, order and organization, and proper security. Each LEA shall develop protocols that define a set of discipline strategies and constructs that ensure that students and adults make positive behavioral choices and that are conducive to a safe and nurturing environment that promotes academic success.*<br>• *C. The model master plan for improving behavior and discipline within the schools and the school master plans required of city, parish, and other local public school boards by this Section shall not prohibit a teacher from removing a pupil from the classroom for disciplinary reasons in accordance with the provisions of R.S. 17:416. D. (1) The school master plans required of city, parish, and other local public school boards by this Section shall make provision for pre-service and ongoing grade appropriate classroom management training for teachers, principals, and other appropriate school personnel regarding positive behavioral supports and reinforcement, conflict resolution, mediation, cultural competence, restorative practices, guidance and discipline, and adolescent development.*
**Social emotional learning (SEL)**	**SE3**	Programs and Services (S/D)	Addresses social-emotional learning (SEL) or character education through development of standards, professional development or classroom instruction.<br><br>**Aligns with NASBE State School Health Policies Database, Curriculum and Instruction, Emotional, Social and Mental Health Education	**0**	Not addressed
				**1**	State law, regulation, or district policy encourages districts to integrate social-emotional learning content or character education into the school curriculum.<br>Standards related to social-emotional development without explicit program requirements are not sufficient for a (1) or (2) code.<br><br>**Example**<br><br>*A. The board of education of every school district in this state*

Variable		Domain	Measure	Score	Brief Description
					may develop and implement a comprehensive program for character education
				**2**	State law, regulation, or district policy requires districts to integrate social-emotional learning content or character education into the school curriculum.<br><br>**Example**<br>*(a) The course of instruction in all public schools shall include character education to help each student develop positive values and improve student conduct*
**Cultural competency**	**SE4**	Staff Qualifications and Professional Development	Incorporates instruction on <u>cultural competency</u> into <u>professional development and teacher education programs</u>.	**0**	State law, regulation, or district policy does not require or encourage incorporation of instruction on cultural competency into professional development or educator training programs; may reference cultural competency as part of educator professional standards.<br><br>**Example**<br>*Effective educational leaders strive for equity of educational opportunity and culturally responsive practices to promote each student's academic success and well-being.*
				**1**	State law, regulation, or district policy encourages districts to provide professional development for school personnel on cultural competency.<br><br>References to cultural competency or teaching of diverse students as part of educator professional standards are not sufficient for a (1) or (2) coding. Must included explicit requirements to deliver cultural competency content as part of pre-service or in-service training.<br><br>**Example**<br>*The department shall develop and make available to school corporations and nonpublic schools materials that assist teachers, administrators, and staff in a school in developing cultural competency for use in providing professional and staff*

Variable		Domain	Measure	Score	Brief Description
					development programs.
				**2**	State law, regulation, or district policy requires districts to provide professional development for school personnel on cultural competency.<br><br>**Example**<br>*The board of trustees of each school district and the governing body of each charter school shall ensure that the teachers and administrators employed by the school district or charter school have access to high-quality, ongoing professional development training. 4. The cultural competency required to meet the social, emotional and academic needs of certain categories of pupils enrolled in the school, including, without limitation, pupils who are at risk, pupils who are English learners, pupils with disabilities and gifted and talented pupils.*
**Early warning and intervention**	**SE5**	Programs and Services (S/D)	Establishes early warning and intervention systems to address truancy and chronic absenteeism through the provision of non-academic supports.	**0**	State law, regulation, or district policy does not address early warning and intervention services or is limited to provisions requiring notification and transfer to law enforcement.<br><br>**Example**<br>*A. The attendance officer may enforce the law relating to:*<br>*1. School attendance of children between the ages of six and sixteen years…*<br>*B. The attendance officer may…*<br>*Report a violation of a law specified in subsection A of this section to the local law enforcement agency and request an investigation of the violation. The law enforcement agency shall, when sufficient cause exists, refer the matter for prosecution.*
				**1**	State law, regulation, or district policy encourages or requires districts to address truancy or chronic absenteeism through use of early warning, parent conferencing and parental sanctions.<br><br>**Example**

Variable		Domain	Measure	Score	Brief Description
					(2) If a child is repeatedly absent from school without valid excuse, or is failing in schoolwork or gives evidence of behavior problems, and attempts to confer with the parent or other person in parental relationship to the child fail, the superintendent of schools, or the intermediate superintendent in a district which does not employ a superintendent, may request the attendance officer to notify the parent or other person in parental relationship by registered mail to come to the school or to a place designated at a time specified to discuss the child's irregularity in attendance, failing work, or behavior problems with the proper school authorities.
				**2**	State law, regulation, or district policy encourages or requires districts to address truancy or chronic absenteeism through the provision of comprehensive student support services.<br><br>**Example**<br><br>*(1) Each school district shall adopt policies to identify students who are at risk of suspension or expulsion from school. Students identified may include those who are truant, who have been or are likely to be declared habitually truant, or who are likely to be declared habitually disruptive. The school district shall provide students who are identified as at risk of suspension or expulsion with a plan to provide the necessary support services to help them avoid expulsion.*
**Bullying, harassment, and intimidation - policy**	**SE6**	Policy or Plan Requirements	Encourages or requires school districts to adopt a bullying, harassment, and intimidation prevention policy<br><br>**Aligns with NASBE State School Health Policies Database, Health Promoting Environment, Bullying, Harassment, and Hazing	**0**	Not addressed
				**1**	State law, regulation, or district policy requires districts to adopt anti-bullying policies addressing some U.S. Department of Education-recommended policy requirements.<br>• Prohibition: statement prohibiting bullying and cyberbullying in schools.<br>• Definition: detailed description of prohibited behavior<br>• Scope: statement of where the policy applies (e.g., on school grounds, at school-sponsored activities, etc.)<br>• Reporting and investigations requirements: required procedures for reporting and investigation of alleged bullying incidents.<br>• Consequences for students who bully: disciplinary sanctions for students who bully.<br>• Communication of policy: statements regarding how

Variable		Domain	Measure	Score	Brief Description
					the policy is publicized communicated to members of the school community, including students, teachers, and parents or family members.<br>• Review and accountability provisions: provisions requiring districts to periodic review and update policy content, and/or submit policies to the state for review and approval.<br><br>**Example**<br>*(a) By July 1, 2007, each school district shall adopt a policy that prohibits the harassment, intimidation, or bullying of any student. Each school district shall share this policy with parents or guardians, students, volunteers, and school employees.*<br>*(b) The policy must be adopted through the standard policy-making procedure for each district that includes the opportunity for participation by parents or guardians, school employees, volunteers, students, administrators, and community representatives. The policy must emphasize positive character traits and values, including the importance of civil and respectful speech and conduct, and the responsibility of students to comply with the district's policy prohibiting harassment, intimidation, or bullying. The policy must also include provisions for an appropriate punishment schedule up to and including expulsion and reporting of criminal activity to local law enforcement authorities. School employees, volunteers, students, and administrators shall adhere to this policy.*
				**2**	State law, regulation, or district policy requires districts to adopt comprehensive anti-bullying policies addressing all U.S. Department of Education-recommended policy requirements.<br><br>• Prohibition: statement prohibiting bullying and cyberbullying in schools.<br>• Definition: detailed description of prohibited behavior<br>• Scope: statement of where the policy applies (e.g., on school grounds, at school-sponsored activities, etc.)<br>• Reporting and investigations requirements: required procedures for reporting and investigation of alleged bullying incidents.

Variable		Domain	Measure	Score	Brief Description
					• Consequences for students who bully: disciplinary sanctions for students who bully.<br>• Communication of policy: statements regarding how the policy is publicized communicated to members of the school community, including students, teachers, and parents or family members.<br>• Review and accountability provisions: provisions requiring districts to periodic review and update policy content, and/or submit policies to the state for review and approval.<br><br>**Example**<br>*See New Jersey bullying statutes 18A:37.13 through 18A:37-32.1*
**Bullying, harassment, and intimidation – professional development**	**SE7**	Staff Qualifications and Professional Development	Incorporates instruction on bullying prevention, intervention, and response into professional development and teacher education programs<br><br>**Aligns with NASBE State School Health Policies Database, Health Promoting Environment, Bullying, Harassment, and Hazing	**0**	Not addressed
				**1**	State law, regulation, or district policy encourages districts to provide professional development for school personnel on bullying prevention and response or requires districts to review district policy content.<br><br>**Example**<br>*A school corporation shall provide training to the school corporation's employees and volunteers who have direct, ongoing contact with students concerning the school's bullying prevention and reporting policy adopted under IC 20-33-8-13.5.*
				**2**	State law, regulation, or district policy requires districts to provide professional development for school personnel on bullying prevention and response.<br><br>**Example**<br>*(2) School districts and charter schools shall provide ongoing professional development to build skills of all school staff members to prevent, identify and respond to harassment, intimidation and bullying. The state board shall promulgate rules regarding the content of the professional development required by this subsection.*

Variable		Domain	Measure	Score	Brief Description
**Bullying, harassment, and intimidation – enumerated/ protected groups**	**SE9**	Program Requirement	Contains <u>explicit protections from harassment or discrimination</u> for vulnerable populations (e.g. LGBTQ youth)	**0**	Not addressed
				**1**	State law, regulation, or district policy prohibits bullying based on personal characteristics without enumerating protected classes or limits coverage to protected groups.<br><br>**Example**<br>*(2) Harassment. — A continuous pattern of intentional behavior that takes place on school property, on a school bus, or at a school-sponsored function including, but not limited to, written, electronic, verbal, or physical acts that are reasonably perceived as being motivated by any characteristic of a student, or by the association of a student with an individual who has a particular characteristic, if the characteristic falls into one of the categories of personal characteristics contained in the model policy adopted by the department or by a local board.*
				**2**	State law, regulation, or district policy enumerates protected classes including explicit protections for LGBTQ youth.<br><br>**Example**<br>*Each plan shall recognize that certain students may be more vulnerable to becoming a target of bullying or harassment based on actual or perceived differentiating characteristics…the plan shall include the specific steps that each school district, charter school, non-public school, approved private day or residential school and collaborative school shall take to support vulnerable students and to provide all students with the skills, knowledge and strategies needed to prevent or respond to bullying or harassment.*
**Hazing**	**SE10**	Policy or Plan Requirements	<u>Prohibits hazing behavior and establishes policies to prevent and respond to hazing</u> in K-12 schools	**0**	Not addressed in K-12 school settings.
				**1**	State law, regulation, or district policy prohibits hazing as a violation of student codes of conduct without requiring districts to adopt hazing policies.<br><br>**Example**<br>*1a. A person commits an act of hazing when the person*

Variable		Domain	Measure	Score	Brief Description
					intentionally or recklessly engages in any act or acts involving forced activity which endanger the physical health or safety of a student for the purpose of initiation or admission into, or affiliation with, any organization operating in connection with a school, college, or university….3. A person who commits an act of hazing which causes serious bodily injury to another is guilty of a serious misdemeanor.
				**2**	State law, regulation, or district policy requires districts to adopt hazing policies.<br><br>**Example**<br>*A. Every public educational institution in this state shall adopt, post and enforce a hazing prevention policy. The hazing prevention policy shall be printed in every student handbook for distribution to parents and students. Each hazing prevention policy shall include…*
**Dating violence and sexual assault prevention**	**SE11**	Policy or Plan Requirements	Encourages or requires districts to adopt dating violence and sexual assault prevention policies.	**0**	Not addressed
				**1**	State law, regulation, or district policy encourages districts to adopt dating violence policies.<br><br>**Example**<br>*A school district governing board may prescribe and enforce policies and procedures to address incidents of dating abuse involving students at school that may be based on a model dating abuse policy.*
				**2**	State law, regulation, or district policy requires districts to adopt dating violence policies.<br><br>**Example**<br>*(1) Each district school board shall adopt and implement a dating violence and abuse policy. The policy shall:(a) Prohibit dating violence and abuse by any student on school property, during a school-sponsored activity, or during school-sponsored transportation.*<br>*(b) Provide procedures for responding to such incidents of dating violence or abuse, including accommodations for*

Variable		Domain	Measure	Score	Brief Description
					students experiencing dating violence or abuse.<br>*(c) Define dating violence and abuse and provide for a teen dating violence and abuse component in the health education curriculum, according to s. 1003.42(2)(n), with emphasis on prevention education. (d) Be implemented in a manner that is integrated with a school district's discipline policies. (2) Each district school board shall provide training for teachers, staff, and school administrators to implement this section.*
**Limits on exclusion**	**SE13**	Prohibitions or Restrictions	Limits use of zero tolerance policies that limit administrator discretion except when in accordance with federal law; limits use of suspension and/or expulsion based on certain conditions.	**0**	State law, regulation, or district policy includes zero tolerance provisions for discretionary offenses (e.g., offenses not governed by federal law, such as firearm possession) and does not place limits or restrictions on use of exclusionary discipline.<br><br>**Example**<br><br>*The board of education of any school district may suspend or expel, or by regulation authorize any certificated employee or committee of certificated employees to suspend or expel, any pupil guilty of any of the following:*<br>*(a) Willful violation of any published regulation for student conduct adopted or approved by the board of education;*<br>*(b) conduct which substantially disrupts, impedes or interferes with the operation of any public school;*<br>*(c) conduct which endangers the safety of others or which substantially impinges upon or invades the rights of others at school, on school property, or at a school supervised activity…*
				**1**	State law, regulation, or district policy discourages district use of zero tolerance approaches and encourages school administrator discretion in determining sanctions for violations of school policy.<br><br>**Example**<br><br>*When revising the prescribed consequences for violation of the student code of conduct pursuant to subsection 15, paragraph C, a school board shall consider districtwide disciplinary policies that: (b) Focus on positive and restorative interventions that are consistent with evidence-based practices rather than set punishments for specific behavior and avoid so-called zero-tolerance practices unless*

Variable		Domain	Measure	Score	Brief Description
					specifically required by federal or state laws, rules or regulations. Allow administrators to use their discretion to fashion appropriate discipline that examines the circumstances pertinent to the case at hand.
				**2**	State law, regulation, or district policy requires districts to limit use of out-of-school suspension and expulsion based on factors such as student age or offense type.<br><br>**Example**<br>*(2) Except as provided in Section 48910, a pupil enrolled in kindergarten or any of grades 1 to 3, inclusive, shall not be suspended for any of the acts enumerated in this subdivision, and this subdivision shall not constitute grounds for a pupil enrolled in kindergarten or any of grades 1 to 12, inclusive, to be recommended for expulsion*<br>*(v) For a pupil subject to discipline under this section, a superintendent of the school district or principal may use his or her discretion to provide alternatives to suspension or expulsion that are age appropriate and designed to address and correct the pupil's specific misbehavior as specified in Section 48900.5.*<br><br>*(w) It is the intent of the Legislature that alternatives to suspension or expulsion be imposed against a pupil who is truant, tardy, or otherwise absent from school activities.*<br><br>*(b) In general. -- (1) Except as provided in paragraph (2) of this subsection, a student enrolled in a public prekindergarten program, kindergarten, first grade, or second grade may not be suspended or expelled from school. (2) A student described under paragraph (1) of this subsection may only be:(i) Expelled from school if required by federal law; or (ii) Suspended for not more than 5 school days if the school administration, in consultation with a school psychologist or other mental health professional, determines that there is an imminent threat of serious harm to other students or staff that cannot be reduced or eliminated through interventions and supports.*
**Alternatives to**	**SE14**	Programs and	Encourages use of <u>restorative justice, restorative</u>	**0**	Not addressed

Variable		Domain	Measure	Score	Brief Description
**exclusion**		Services	practices, or other non-punitive discipline approaches as alternatives to exclusion or school removal.	**1**	State law, regulation, or district policy encourages districts to use alternatives to out-of-school suspension or expulsion, such as, in-school suspension, behavioral interventions, or restorative practices.<br>**Example(s)**<br>(*1) A school board or its designee shall consider using restorative practices as an alternative or in addition to suspension or expulsion under this act. If a school board or its designee suspends or expels a pupil under this act, the school board or its designee shall consider using restorative practices in addition to suspension or expulsion. If a school board or its designee decides not to suspend or expel a pupil for a disciplinary issue, the school board or its designee shall consider using restorative practices to address the disciplinary issue.*
				**2**	State law, regulation, or district policy requires districts to use alternatives to out-of-school suspension or expulsion, such as, in-school suspension, behavioral interventions, or restorative practices.<br>**Example**<br>*(II) In creating and enforcing a school conduct and discipline code pursuant to subparagraph (I) of this paragraph (a), each school district board of education, on and after August 1, 2013, shall: (B) Include plans for the appropriate use of prevention, intervention, restorative justice, peer mediation, counseling, or other approaches to address student misconduct, which approaches are designed to minimize student exposure to the criminal and juvenile justice system.*<br>*When revising the prescribed consequences for violation of the student code of conduct pursuant to subsection 15, paragraph C, a school board shall consider districtwide disciplinary policies that: (a) Focus on positive interventions and expectations and avoid focusing exclusively on unacceptable student behavior. For the purpose of this subsection, "positive interventions" means instructional and environmental supports that are designed to teach students prosocial alternatives to problem behaviors with high rates of*

**Variable**		Domain	Measure	Score	Brief Description
					positive feedback; Focus on positive and restorative interventions that are consistent with evidence-based practices rather than set punishments for specific behavior and avoid so-called zero-tolerance practices unless specifically required by federal or state laws, rules or regulations. For the purpose of this paragraph, “restorative interventions” means school practices that are designed to strengthen relationships, improve the connection to school and promote a strong sense of accountability and that help students learn from their mistakes, understand the impact of their actions on others and find opportunities to repair the harm they have caused through their misbehavior;
**School Resource Officer (SRO) training**	**SE17**	Staff Qualifications and Professional Development	Requires training for school for resource officers (SRO) addressing issues such as child and adolescent development, mental health crisis interventions, positive behavioral intervention, and de-escalation techniques.	**0**	Not addressed
				**1**	State law, regulation, or district policy encourages SROs to complete specialized training to meet the safety needs of the school environment or requires training in selected schools only.<br>Specialized training may include training addressing adolescent development, cultural competency, de-escalation techniques, or other factors specific to working within a school setting. Excludes firearms training.<br>**Example**<br>*Development. -- The Maryland Police Training Commission, in consultation with the Department, shall develop a cultural competency model training curriculum for law enforcement officers assigned to public schools. (b) Contents. -- (1) The cultural competency model training curriculum shall teach behaviors, attitudes, and policies that enable law enforcement officers to understand, communicate with, and effectively interact with the individuals, organizations, and institutions in the community in which the public school to which a law enforcement officer is assigned is located. (2) The cultural competency model training curriculum shall include:(i) Personal exposure to the individuals, organizations, and institutions within the assigned community; and (ii) Knowledge of government and community services available to help prevent juvenile arrests. (c) Officer assigned to patrol school. -- A law enforcement officer who is assigned to patrol a school building or school grounds is encouraged to*

Variable		Domain	Measure	Score	Brief Description
					complete the cultural competency model training curriculum established under subsection (b) of this section before the law enforcement officer begins an assignment in a public school.<br>*(b) The commission by rule shall require a school district peace officer or a school resource officer who is commissioned by or who provides law enforcement at a school district with an enrollment of 30,000 or more students to successfully complete an education and training program*
				**2**	State law, regulation, or district policy requires SROs to complete specialized training to meet the safety needs of the school environment<br>**Example**<br>*(f) Any school police officer first employed by a K-12 public school district or California Community College district after July 1, 1999, shall successfully complete a basic course of training as prescribed by subdivision (a) before exercising the powers of a peace officer. A school police officer shall not be subject to this subdivision while participating as a trainee in a supervised field training program approved by the Commission on Peace Officer Standards and Training. (g) The commission shall prepare a specialized course of instruction for the training of school peace officers, as defined in Section 830.32, to meet the unique safety needs of a school environment. This course is intended to supplement any other training requirements.*
**Limits on referrals to law enforcement**	**SE18**	Prohibitions or Restrictions	Limits referrals to law enforcement for school-based offenses beyond federally-mandated reporting requirements (e.g., removing mandatory reporting to law enforcement)	**0**	State law, regulation, or district policy requires districts to refer students to local law enforcement for any offense that may be a violation of the criminal code.<br>**Example**<br>*(b) It is the intention of the Legislature by passage of the section to require principals, teachers and other school employees of public elementary, junior and senior high schools to make reports of violent disruptive incidents occurring on school property*<br>*(c) Principals shall file a report within 72 hours with the superintendent of education of any incident of which they have knowledge. A copy of the report shall also be furnished members of the school board and the county sheriff by the superintendent of education…*

Variable		Domain	Measure	Score	Brief Description
					(e) Any superintendent of education, principal, teacher, or employee who violates the provisions of this section by failure to file a required report shall be guilty of a Class C misdemeanor.<br><br>*(b) The principal shall notify appropriate law enforcement officials when any person violates local board of education policies concerning drugs, alcohol, weapons, physical harm to a person, or threatened physical harm to a person.*
				**1**	State law, regulation, or district policy authorizes districts to refer students to local law enforcement or does not address law enforcement reporting other than for federally-mandated reporting for selected offense types.<br><br>**Example**<br><br>*If a teacher knows or has reason to believe that a student is using, is in possession of, or is delivering alcohol or a controlled substance while the student is on school property, involved in a school-related activity, or in attendance at a school-sponsored event, the teacher shall notify the student's principal… This section does not prevent a teacher or any other school employee from reporting to a law enforcement agency any violation of law occurring on school property, at a school-related activity, or at a school-sponsored event.*
				**2**	State law, regulation, or district policy requires districts to establish formal procedures governing referrals to local law enforcement.<br><br>**Example**<br><br>*The student code of conduct must: (g) Establish guidelines and criteria concerning the appropriate circumstances when the superintendent or the superintendent's designee may provide information to the local police or other appropriate law enforcement authorities regarding an offense that involves violence committed by any person on school grounds or other school property*
**Equitable discipline**	**SE19**	Program Requirements	Implements strategies to identify and address racial and ethnic disparities in student discipline,	**0**	Not addressed or only addressed in relation to special education.

**Variable**		Domain	Measure	Score	Brief Description
**practices**			including disaggregating discipline data reports.	**1**	State law, regulation, or district policy requires districts to monitor disproportionality in discipline practices within general education populations.<br><br>**Example**<br><br>*(a) Each local board of education shall file an annual report…with the Department of Education regarding disciplinary and placement actions taken during the prior school year…(b) For each category of disciplinary or placement action… the local board shall provide the following information: the number of students subject to the type of disciplinary or placement action; the age and grade level of such students; such students' race and gender; and the number of students subject to the type of disciplinary action who were eligible for free or reduced price lunches under federal guidelines.*
				**2**	State law, regulation, or district policy requires districts to monitor and remediate disproportionality in discipline practices within general education populations.<br><br>**Example**<br><br>(a) As used in this section, "exclusionary disciplinary actions" means out-of-school suspension and expulsion. (b) (1) Annually, the Department of Education shall report at the school, school district, and state level the following data concerning exclusionary disciplinary actions, in-school suspensions, and corporal punishment:*(d) The department shall:*<br>*(1) Provide school districts with resources for the best practices in effective school discipline; and (2) Annually communicate to school districts: (A) The availability of and how to access the data listed in subsection (b) of this section; and (B) How to access the resources listed in subdivision (d)(1) of this section.*

Physical Environment

Variable		Domain	Measure	Score	Brief Description
**School emergency operations plans (EOP) – plan or policy**	**PE1**	Policy or Plan Requirements	Encourages or requires districts to establish formal plans for responding to crises or emergency events.<br><br>**Aligns with NASBE State School Health Policies Database, Health Promoting Environment, Crisis Management/Emergency Response	**0**	Not addressed
				**1**	State law, regulation, or district policy requires districts to develop school emergency operations plans (EOPs) that do not include procedures for plan review and update.<br><br>**Example**<br>*To the extent funds are available, school districts shall annually review and update safe school plans in collaboration with local emergency response agencies.*
				**2**	State law, regulation, or district policy requires districts to develop school emergency operations plans (EOPs) that include procedures for plan review and update.<br><br>**Example**<br>*A school crisis management and response plan shall be prepared by each public school principal jointly with local law enforcement, fire, public safety, and emergency preparedness officials…Each public school principal, jointly with local law enforcement, fire, public safety, and emergency preparedness officials, shall review the plan at least once annually and shall revise the plan as necessary.*
**School emergency operations plans (EOP) – practice drills**	**PE3**	Program Requirements	Encourages or requires districts to plan and implement multi-hazard practice drills and exercises.<br><br>**Aligns with NASBE State School Health Policies Database, Health Promoting Environment, Crisis Management/Emergency Response	**0**	Not addressed
				**1**	State law, regulation, or district policy requires districts to implement multi-hazard practice drills (e.g., fire, lockdown, active shooter, or evacuation drills) without requiring inter-agency coordination.<br><br>**Example**<br>*The drill shall include the actual use thereof, and the complete removal of the pupils and teachers, in an expeditious and orderly manner, by means of fire-escapes and exits, from the*

Variable		Domain	Measure	Score	Brief Description
					building to a place of safety on the ground outside. (3) The chief school administrator shall notify and request assistance from the local law enforcement agency and emergency management agency before conducting the school security drill.
				**2**	State law, regulation, or district policy requires districts to implement multi-hazard practice drills (e.g., fire, lockdown, active shooter, or evacuation drills) and encourages or requires inter-agency coordination.<br><br>**Example**<br>*Notwithstanding other provisions of this section, in all schools or buildings used for educational purposes through the twelfth grade by six (6) or more persons for four (4) or more hours per day or more than twelve (12) hours per week, there shall be not less than one emergency egress drill conducted every month the facility is in session with all occupants of the building participating in said drill. One additional emergency egress drill shall be conducted in buildings that are not open on a year- round basis within the first (1st) thirty (30) days of operation. At least one out of every four (4) emergency egress drills or rapid dismissals shall be obstructed by means of which at least one or more exits and stairways in the school building are blocked off or not used. In addition, there shall be two (2) evacuation drills and two (2) lockdown drills. Evacuation drills shall be designed and conducted for use when conditions outside the school building are safer than conditions inside the building. Lockdown drills shall be designed and conducted for use to protect school building occupants from potential dangers in the building, and one shall be held in September and one in January, and in conjunction with the local police whenever possible.*
**Firearms/ Weapons on School Campuses**	**PE4**	Prohibitions or Restrictions	<u>Prohibits student possession of firearms or other weapons</u> on school campuses in compliance with federal Gun-Free Schools Act mandates and imposes fair and reasonable consequences for violations (e.g., due process, opportunities for alternative placement).	**0**	State law, regulation, or district policy does not comply with minimum requirements of the federal Gun-Free Schools Act (GFSA).<br><br>• Does not include minimum one-year expulsion with modification on a case-by-case basis.<br>• Does not include mandatory referral to law enforcement.

Variable		Domain	Measure	Score	Brief Description
				**1**	State law, regulation, or district policy complies with minimum requirements of the federal Gun-Free Schools Act (GFSA).<br><br>**Example**<br><br>*(d) Each school district shall adopt a policy providing for the (1) referral to law enforcement authorities of students who violate AS 11.61.210(a)(8)*
				**2**	State law, regulation, or district policy complies with minimum requirements of the federal Gun-Free Schools Act (GFSA) and adds student supports and protections.<br><br>**Example(s)**<br><br>• *The discipline of any public school student for violating a school policy relating to the possession or use of alcohol, drugs or weapons not described in § 16-21-18 of this chapter, shall be imposed on a case-by-case basis pursuant to guidelines developed and promulgated by the school committee for that district. The guidelines and any discipline imposed shall take into account the nature and circumstances of the violation and the applicability of any federal laws governing students with disabilities.*<br>• *2m. The school board shall commence proceedings under subd. 3. and expel a pupil from school for not less than one year whenever it finds that the pupil, while at school or while under the supervision of a school authority, possessed a firearm, as defined in 18 USC 921 (a) (3)...Prior to expelling a pupil, the school board shall hold a hearing.*
**Tobacco-free environments**	**PE5**	Policy or Plan Requirements	Prohibits tobacco use on school campuses and requires districts to adopt comprehensive tobacco-free schools policies.<br><br>**Aligns with NASBE State School Health Policies Database, Health Promoting Environment, Tobacco Use	**0**	Not addressed or limited to prohibiting student possession or use of tobacco or tobacco products.<br><br>**Example**<br><br>*(1) An individual may not use a tobacco product in a public school building or on public school property. (2) Subsection (1) does not apply to the use of a tobacco product in a classroom or on other school property as part of a lecture, demonstration, or educational forum sanctioned by a school administrator or faculty member concerning the risks associated with use of a tobacco product. (3) The principal of*

Variable		Domain	Measure	Score	Brief Description
					an elementary or secondary school, or the principal's designee, may enforce this section. (4) A violation of this section is subject to the penalties provided in 50-40-115. (5) For the purposes of this section, the following definitions apply: (a) "Public school building" or "public school property": (i) means public land, fixtures, buildings, or other property owned or occupied by an institution for the teaching of minor children that is established and maintained under the laws of the state of Montana at public expense; and (ii) includes school playgrounds, school steps, parking lots, administration buildings, athletic facilities, gymnasiums, locker rooms, and school buses. (b) "Tobacco product" means a substance intended for human consumption that contains tobacco, including cigarettes, cigars, snuff, smoking tobacco, and smokeless tobacco.
				**1**	State law, regulation, or district policy prohibits any smoking or use of tobacco products on school grounds when students are present and may require districts to adopt tobacco-free school policies that address some CDC components.<br><br>• An explanation of the rationale for preventing tobacco use (i.e., tobacco is the leading cause of death, disease, and disability)<br>• Prohibitions against tobacco use by students, all school staff, parents, and visitors on school property, in school vehicles, and at school-sponsored functions away from school property<br>• Prohibitions against tobacco advertising in school buildings, at school functions, and in school publications<br>• A requirement that all students receive instruction on avoiding tobacco use<br>• Provisions for students and all school staff to have access to programs to help them quit using tobacco<br>• Procedures for communicating the policy to students, all school staff, parents or families, visitors, and the community<br>• Provisions for enforcing the policy<br><br>**Example**<br><br>*(a) Tobacco and tobacco products are prohibited in public school and public charter school buildings, grounds, parking*

**Variable**		Domain	Measure	Score	Brief Description
					lots, parking garages, playing fields, school buses and other vehicles, and at off-campus, school-sponsored events.<br>*(b) For a public charter school located in a mixed-use facility, the requirements of subsection (a) of this section shall apply only to the buildings, grounds, parking lots, garages, and fields under the control of the public charter school.*
				**2**	State law, regulation, or district policy requires districts to adopt comprehensive tobacco-free school policies that address most or all CDC components.<br><br>• An explanation of the rationale for preventing tobacco use (i.e., tobacco is the leading cause of death, disease, and disability)<br>• Prohibitions against tobacco use by students, all school staff, parents, and visitors on school property, in school vehicles, and at school-sponsored functions away from school property<br>• Prohibitions against tobacco advertising in school buildings, at school functions, and in school publications<br>• A requirement that all students receive instruction on avoiding tobacco use<br>• Provisions for students and all school staff to have access to programs to help them quit using tobacco<br>• Procedures for communicating the policy to students, all school staff, parents or families, visitors, and the community<br>• Provisions for enforcing the policy<br><br>Must include provisions related to smoking cessation for coding as a (2).<br>**Example**<br>*…to improve the health of students and school personnel, each school district and charter school in Delaware shall have a policy which at a minimum: 1.1 Prohibits the use of or distribution of tobacco products in school buildings, on school grounds, in school leased or owned vehicles, even when they are not used for student purposes, and at all school affiliated functions. 1.2 Includes procedures for communicating the policy to students, school staff, parents, guardians or Relative Caregivers, families, visitors and the community at large.*<br>*1.3 Makes provisions for or refers individuals to voluntary*

Variable		Domain	Measure	Score	Brief Description
					cessation education and support programs that address the physical and social issues associated with nicotine addiction.
**Alcohol and drug use – intervention programs**	**PE7**	Programs and Services	Encourages or requires districts to implement school-based alcohol and drug counseling referral, intervention or treatment programs for students identified with substance use disorders.	**0**	Not addressed
				**1**	State law, regulation, or district policy encourages districts to implement school-based alcohol and drug referral, intervention or treatment programs for students with substance use disorders.<br>**Example**<br>*The board of education of every school district may provide for programs which are designed to assist pupils at all grade levels in the identification, examination, prevention and resolution of alcohol and drug abuse problems which may affect the ability of such pupils to satisfactorily benefit from attendance at school.*
				**2**	State law, regulation, or district policy requires districts to implement school-based alcohol and drug referral, intervention or treatment programs for students with substance use disorders.<br>**Example**<br>*In compliance with written school policy adopted by a school board, the school board may require that a student who has been determined to be in violation of school rules governing substance abuse or alcohol or drug possession participate in a substance abuse assessment, education or support group service offered by the school. The school board shall provide for notice to the parents or legal guardian of a student required to participate in such services. If the school board elects to do so, it may request a parent or legal guardian to participate in the services.*
**Corporal punishment**	**PE8**	Prohibitions or Restrictions	Prohibits the use of corporal punishment in schools.	**0**	State law, regulation, or district policy permits the use of corporal punishment for disciplinary purposes.<br>**Example(s)**

Variable		Domain	Measure	Score	Brief Description
				1	• *Any teacher or school principal may use corporal punishment in a reasonable manner against any pupil for good cause in order to maintain discipline and order within the public schools.*<br>• *The district school board may prohibit the use of corporal punishment, if the district school board adopts or has adopted a written program of alternative control or discipline.*
				1	State law, regulation, or district policy neither permits nor prohibits the use of corporal punishment for disciplinary purposes.<br>**Example(s)**<br>• *Superintendents, principals, supervisors, and teachers and their aids and assistants, have the authority, to use the physical force that is reasonable and necessary for supervisory control over students.*<br>• *Policies and procedures for the use of acts of reasonable and appropriate physical intervention or force in dealing with disruptive students; except that no board shall adopt a discipline code that includes provisions that are in conflict with the definition of child abuse in section 18-6-401 (1), C.R.S., and section 19-1-103 (1), C.R.S.;*
				2	State law, regulation, or district policy prohibits the use of corporal punishment for disciplinary purposes without exception.<br>**Example**<br>*The use of corporal punishment in any form is strictly prohibited in and during all aspects of the public school environment or school activities.*
**Restraint and seclusion**	**PE9**	Prohibitions or Restrictions	Limits use of restraint and seclusion except under specified conditions (e.g. immediate threats to safety).	**0**	Not addressed
				**1**	State law, regulation, or district policy requires districts to adopt policies that limit use of restraint and seclusion without addressing comprehensive student protections.<br>**Example**

Variable		Domain	Measure	Score	Brief Description
					The restraint or seclusion technique shall be used only by school personnel who are trained in the safe and effective use of restraint and seclusion techniques unless an emergency situation does not allow sufficient time to summon trained personnel.
				**2**	State law, regulation, or district policy requires districts to adopt policies limiting use of restraint and seclusion that include comprehensive student protections, including requirements for school personnel to be trained in safe restraint procedures.<br><br>Comprehensive student protections may include: restrictions on use to cases involving threats to health and safety, training requirements, mandatory reporting, and required debriefing following incidents.<br><br>**Example**<br><br>*(a) The department shall approve crisis intervention training programs for schools, which shall include training in (1) evidence-based techniques that have been shown to be effective in the prevention of restraint and seclusion of students;*<br>*(2) evidence-based techniques shown to be effective in keeping school personnel and students safe when imposing physical restraint or seclusion of students;*<br>*(3) evidence-based skills related to positive behavior supports, conflict prevention, understanding antecedents, de-escalation, and conflict management;*<br>*(4) first aid and cardiopulmonary resuscitation; and*<br>*(5) applicable policies and procedures.*<br>*(b) The governing body of a school shall ensure that a sufficient number of school employees receives periodic training in an approved crisis intervention program to meet the needs of the school population.*
**Safety and Security - partnerships**	**PE11**	Partnerships	Authorizes Memorandums of Understanding (MOUS), partnerships or other agreements between schools and local law enforcement to place School Resource Officers or security personnel on school campuses.	**0**	Not addressed.
				**1**	State law, regulation, or district policy authorizes the placement of local law enforcement or security personnel on school campuses through MOUs or partnership agreements.

Variable		Domain	Measure	Score	Brief Description
					**Example**<br>*A local board of education may employ persons as school security personnel or contract with a local chief of police or sheriff to employ school resource officers. A local board of education may allow any person employed by the board as school security personnel or as a school resource officer to carry a firearm while on duty if the employee satisfies all of the following qualifications:*
				**2**	State law, regulation, or district policy authorizes the placement of local law enforcement or security personnel and establishes clear guidelines and safeguards regarding the appropriate role of officers on school campuses.<br><br>**Example**<br>*(1) If a school resource officer or other law enforcement officer acting in his or her official capacity on school grounds, in a school vehicle, or at a school activity or sanctioned event arrests a student of the school, the officer shall notify the principal of the school or his or her designee of the arrest within twenty-four hours after the arrest.*
**Internet Safety**	**PE12**	Policy or Plan Requirements	Encourages or requires districts to adopt internet safety policies.	**0**	Not addressed or limited in scope (e.g., sexting only)
				**1**	State law regulation or district policy encourages districts to adopt acceptable Internet use policies or to provide instruction on safe Internet use.<br><br>**Example**<br>*1. Any school district in the state may provide, to pupils in grades kindergarten through twelve, instruction designed to promote the proper and safe use of the internet.*
				**2**	State law, regulation, or district policy requires districts to adopt Internet use policies or to provide instruction on safe Internet use.<br><br>**Example**<br>*(a) (1) Every two (2) years, each director of schools shall file with the commissioner of education an acceptable use policy, approved by the local school board, for the international*

<table>
<tr><th colspan="2">Variable</th><th>Domain</th><th>Measure</th><th>Score</th><th>Brief Description</th></tr>
<tr><td></td><td></td><td></td><td></td><td></td><td>network of computer systems commonly known as the Internet.</td></tr>
<tr><td rowspan="3">Chemical hazards</td><td rowspan="3">PE16</td><td rowspan="3">Prohibitions</td><td rowspan="3">Establishes protections for students by prohibiting use of certain harmful chemicals in schools, such as specific pesticides, and cleaning agents; requires parent notification and documentation of chemical applications.</td><td>0</td><td>Not addressed</td></tr>
<tr><td>1</td><td>State law, regulation, or district policy encourages districts to utilize environmentally-safe chemicals and to reduce chemical exposure in schools.

Example(s)
• The department of elementary and secondary education shall, in consultation with the department of health and senior services, and a panel of interested stakeholders, including cleaning product industry representatives, nongovernmental organizations, and others, establish and amend on an annual basis guidelines and specifications for green cleaning programs, including environmentally sensitive cleaning and maintenance products, paper product purchases, and equipment purchases for cleaning programs.
• A school is not required to adopt an integrated pest management plan. A school board may only notify students, parents, or employees that it has adopted an integrated pest management plan if the plan is a managed pest control program designed to minimize the risk to human health and the environment and to reduce the use of chemical pesticides, and which ranks the district's response to pests</td></tr>
<tr><td>2</td><td>State law, regulation, or district policy requires districts to utilize environmentally-safe chemicals and/or to reduce chemical exposure in schools.

• Includes integrated pest management programs and policies and green cleaning programs.

Example
(a) (1) An environmental programs office is established in the Department of General Services and shall: (D) Establish an integrated pest management program; (E) Test drinking water in public schools for lead and promptly take any remedial</td></tr>
</table>

Variable		Domain	Measure	Score	Brief Description
					action required; (b) The District of Columbia Public Schools shall: (1) Use environmentally friendly cleaning supplies in public schools; provided, that the agency may exhaust its current supply of conventional cleaners; and (1) Use environmentally friendly cleaning supplies in public schools; provided, that the agency may exhaust its current supply of conventional cleaners
**Water quality**	**PE17**	Program Requirement	Establishes water quality programs, potable water standards, and testing protocols to ensure safe drinking water.	**0**	Not addressed
				**1**	State law, regulation, or district policy encourages districts to address water quality in schools.<br><br>**Example**<br>*(a) (1) An environmental programs office is established in the Department of General Services and shall: (D) Establish an integrated pest management program; (E) Test drinking water in public schools for lead and promptly take any remedial action required; (b) The District of Columbia Public Schools shall: (1) Use environmentally friendly cleaning supplies in public schools; provided, that the agency may exhaust its current supply of conventional cleaners; and (1) Use environmentally friendly cleaning supplies in public schools; provided, that the agency may exhaust its current supply of conventional cleaners*
				**2**	State law, regulation, or district policy requires districts to address water quality in schools.<br><br>**Example**<br>*By July 1, 2018, the board of each school district or charter school must adopt the commissioners" model plan or develop and adopt an alternative plan to accurately and efficiently test for the presence of lead in water in school buildings serving prekindergarten students and students in kindergarten through grade 12. To the extent possible, the commissioners shall base the plan on the standards established by the United States Environmental Protection Agency.*
**Air quality**	**PE18**	Program	Encourages or requires districts to establish	**0**	Not addressed

**Variable**		Domain	Measure	Score	Brief Description
		Requirement	**indoor** air quality plans or policies or includes direct mandates for routine inspection and evaluation of indoor air quality in schools.	**1**	State law, regulation, or district policy encourages districts to address air quality issues in schools.<br><br>**Example**<br>*(a) Each LEA is encouraged to conduct an inspection and evaluation program, such as the environmental protection agency's indoor air quality tools for schools program, for its facilities.*
				**2**	State law, regulation, or district policy requires districts to address indoor air quality in schools.<br><br>**Example**<br>*(a) Each local or regional board of education shall adopt and implement an indoor air quality program that provides for ongoing maintenance and facility reviews necessary for the maintenance and improvement of the indoor air quality of its facilities*
