# Supplementary material for: State Laws Matter When It Comes to District Policymaking Relative to the Whole School, Whole Community, Whole Child Framework
Source: J Sch Health. 2020 Nov 12;90(12):907–17. doi: 10.1111/josh.12959 (PMC7702124; doi:10.1111/josh.12959)
Supplement: Supplementary file 2 — Appendix S2. Predicted Mean District Policy Score and Change in Score by Domain Associated with District‐Weighted Mean and Comprehensive State Law Scores [file JOSH-90-907-s002.pdf]

Appendix S2. Predicted Mean District Policy Score and Change in Score by Domain Associated with District-Weighted Mean and Comprehensive State Law Scores

			Unadjusted Models		Adjusted Models	
**Domain (# of Items in Domain)**	**State Score**		Predicted District Score	%age point Δ in District Score	Predicted District Score	%age point Δ in District Score
Community Involvement (3)	Mean	73.8	76.4	-2	76.0	**-3**
	Full	100	74.2		72.6	
Counseling, Psychological, and Social Services (8)	Mean	70.5	49.1	**-13**	49.3	**-7**
	Full	100	36.1		42.6	
Employee Wellness (5)	Mean	15.5	17.3	12	17.6	1
	Full	100	29.3		18.6	
Family Engagement (4)	Mean	60.5	53.5	1	53.2	**16**
	Full	100	54.6		69.3	
Health Education (10)	Mean	69.9	56.5	**-12**	55.9	**-22**
	Full	100	44.7		33.7	
Health Services (10)	Mean	77.6	52.9	**13**	52.6	**11**
	Full	100	65.4		63.9	
Nutrition Environment and Services (6)	Mean	32.0	49.8	7	50.3	-3
	Full	100	56.8		47.1	
Physical Activity and Education (7)	Mean	49.4	56.6	**46**	56.8	**29**
	Full	100	102.2		85.8	
Physical Environment (12)	Mean	80.6	62.1	1	61.5	0
	Full	100	63.5		61.6	
Social and Emotional Climate (14)	Mean	66.8	50.8	**11**	50.5	**11**
	Full	100	61.8		61.9	
Overall (79)	Mean	63.5	52.8	**4**	52.6	**9**
	Full	100	57.0		62.0	

Notes: Items in **bold** reflect statistically significant differences at the $p<.05$ threshold or lower based on the regression models presented in Table 3.
